# Ferroptosis at the nexus of metabolism and metabolic diseases

**DOI:** 10.7150/thno.100080

**Published:** 2024-09-09

**Authors:** Shuangwen Li, Guixiang Zhang, Jiankun Hu, Yan Tian, Xianghui Fu

**Affiliations:** 1 Department of Endocrinology and Metabolism, Department of Biotherapy, Center for Diabetes and Metabolism Research, State Key Laboratory of Biotherapy and Cancer Center, West China Hospital, Sichuan University, Chengdu, 610041, Sichuan, China.; 2 Division of Gastrointestinal Surgery, Department of General Surgery, West China Hospital, Sichuan University, Chengdu, 610041, Sichuan, China.; 3 Gastric Cancer Center, West China Hospital, Sichuan University, Chengdu, 610041, Sichuan, China.; 4 Department of General Surgery, West China Hospital, Sichuan University, Chengdu, 610041, Sichuan, China.

**Keywords:** Redox metabolism, Glucose catabolism, Lipid peroxidation, Ferroptosis cascade responses, Ferroptosis-related therapeutics

## Abstract

Ferroptosis, an iron-dependent form of regulated cell death, is emerging as a crucial regulator of human physiology and pathology. Increasing evidence showcases a reciprocal relationship between ferroptosis and dysregulated metabolism, propagating a pathogenic vicious cycle that exacerbates pathology and human diseases, particularly metabolic disorders. Consequently, there is a rapidly growing interest in developing ferroptosis-based therapeutics. Therefore, a comprehensive understanding of the intricate interplay between ferroptosis and metabolism could provide an invaluable resource for mechanistic insight and therapeutic development. In this review, we summarize the important metabolic substances and associated pathways in ferroptosis initiation and progression, outline the cascade responses of ferroptosis in disease development, overview the roles and mechanisms of ferroptosis in metabolic diseases, introduce the methods for ferroptosis detection, and discuss the therapeutic perspectives of ferroptosis, which collectively aim to illustrate a comprehensive view of ferroptosis in basic, translational, and clinical science.

## Introduction

As an emerging mode of programmed cell death, ferroptosis has attracted tremendous interest in the past decade due to its increasing importance in both physiology and pathology. In principle, ferroptosis is driven by iron-dependent lipid peroxidation, which results from a combinational outcome of aberrant cellular metabolism and imbalanced redox homeostasis. Abnormal metabolism of diverse nutrients, such as glucose, amino acids, lipids, and minerals, may potentiate lipid peroxidation and consequent ferroptosis by increasing the buildup of reactive oxygen species (ROS) or polyunsaturated fatty acids (PUFAs) 1. Correspondingly, many key metabolic regulators, such as adenosine 5'-monophosphate-activated protein kinase (AMPK), mammalian target of rapamycin (mTOR), α-enolase (ENO), and 7-dehydrocholesterol (7-DHC) have been implicated in ferroptosis initiation and progression 2-6. Conversely, several systems that are closely associated with the homeostasis of various amino acids and minerals comprise the resistance mechanisms against ferroptosis, including the system Xc^-^/glutathione (GSH)/glutathione peroxidase 4 (GPX4) system, GTP cyclohydrolase-1 (GCH1)/tetrahydrobiopterin (BH4) pathway, and coenzyme Q10 (CoQ10) 7-9. Therefore, the inception and progression of ferroptosis are an integrated outcome of numerous dysregulated metabolic pathways under the control of both inducible and inhibitory molecules. Additionally, ferroptosis has been observed to exacerbate several pathological processes and human diseases, including obesity, type 2 diabetes (T2D) and its complications, and non-alcoholic fatty liver disease (NAFLD), by further disrupting metabolic homeostasis, amplifying the propagation of ferroptotic death signals, and initiating inflammatory responses 10-13. Consequently, the implementation of ferroptosis-based therapies holds immense promise in catalyzing remarkable strides in the field of disease treatment.

Overall, ferroptosis appears to be a critical nexus for the integration of metabolism, redox biology, and pathophysiology. A better understanding of the interplay between ferroptosis and metabolism may provide new mechanistic insights and potential therapeutic strategies for the prevention and treatment of human diseases.

## The engines of ferroptosis

**Figure 1:**
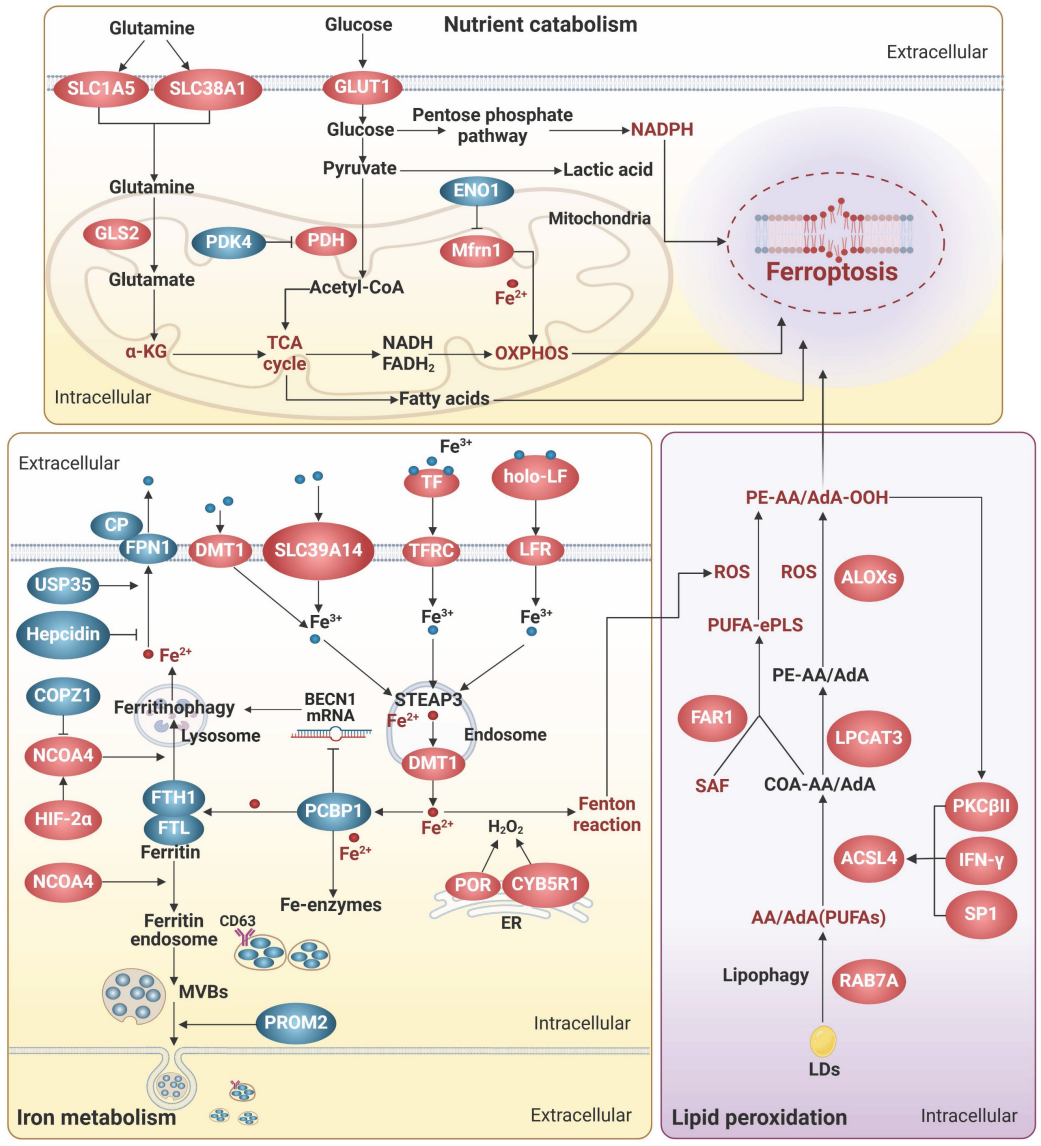
** Metabolic engines for ferroptosis.** Lipid peroxidation plays a central role in the process of ferroptosis. Disruptions in the homeostasis of iron, glucose, amino acids, and lipids result in the accumulation of highly toxic ROS and/or PUFAs. Specifically, dysregulations in iron uptake, transport, storage, utilization, and export can lead to iron overload, which triggers excessive ROS production through the Fenton reaction. Furthermore, iron serves as a vital component of various oxidoreductases that are intricately involved in the redox system. The TCA cycle and OXPHOS interconnect metabolism of glucose, amino acids, and lipid to supply the necessary ROS and PUFAs for ferroptosis. However, the role of nutrient metabolism in ferroptosis extends beyond this. The production of NADPH, the synthesis of oxidoreductases, and the process of lipophagy are associated with ferroptosis. Arrows and vertical bars indicate positive and negative effects, respectively. Created with BioRender.com.

It is widely accepted that labile iron accumulation tends to drive ferroptosis. Meanwhile, perturbations in metabolic pathways of fundamental energy substrates like glucose, lipids, and amino acids could augment the buildup of ROS and trigger the peroxidation of PUFAs, heightening the susceptibility to ferroptosis. In this section, we overview the functions and mechanisms of ferroptosis inducers and effectors, with a focus on the aberrant metabolism of iron, glucose, amino acids, and lipids (**Figure 1**).

### Iron metabolism

Iron homeostasis is a multi-step process involving iron transport, uptake, storage, and export, and is tightly regulated to avoid excess iron accumulation. Excess iron can contribute to ferroptosis by catalyzing the Fenton reaction or by functioning as the active core of oxidoreductases, including the nicotinamide adenine dinucleotide phosphate hydride (NADPH) oxidases (NOXs), xanthine oxidase, arachidonic acid lipoxygenases (ALOXs), and cytochrome P450 enzymes (**Figure 1**).

#### Iron transportation and absorption

Generally, iron absorption is accomplished by two ways: transferrin receptor (TFRC)-mediated endocytosis of iron-loaded transferrin (TF) and direct iron intake mediated by metal transporters. TF and TFRC have been extensively implicated in ferroptosis 14. TF binds to free iron, forms a complex with TFRC on the cell surface, and transports iron via endocytosis. It is worth noting that lactoferrin (LF), another TF family member, can regulate ferroptosis beyond iron transportation. Iron free LF shows a superior anti-ferroptosis property by increasing the expression of GPX4, solute carrier family 7 member 11 (SLC7A11), and nuclear factor erythroid 2-related factor 2 (Nrf2) 15, 16. Moreover, LF orchestrates the function of pivotal proteins within the iron metabolism network. For instance, Fe^2+^ needs to be catalyzed to Fe^3+^ by iron oxidizing enzymes, such as ceruloplasmin, before it can be transported out of the cell by ferritin 1 (FPN1). LF enhances the iron oxidase efficacy of ceruloplasmin, thereby augmenting the exportation of iron 17.

Non-transferrin-bound iron (NTBI) can directly enter cells through metal transporters, such as divalent metal transporter 1 (DMT1), SLC39A14, and SLC39A8. DMT1 functions as the major iron transporter for non-heme iron uptake in most cell types under NTBI conditions, while SLC39A14 and SLC39A8 act primarily in the liver, skeletal muscle, and pancreas 18-21. As cytoplasmic NTBI is unstable and prone to generate free radicals, anachronistic increases in metal transporters may augment the propensity for ferroptosis 19-21. Intriguingly, SLC39A14 and SLC39A8 are also responsible for the transportation of other metal ions, including Zn^2+^ and Mn^2+^, which potentially collaborate with iron to facilitate the process of ferroptosis 22.

#### Iron utilization and storage

Metals are utilized by transferring to their cognate proteins through the mediation of metallochaperones. Frataxin and poly(rC)-binding proteins (PCBPs) are crucial iron chaperones, which aid in delivering iron to iron-containing functional proteins for utilization or to ferritin for storage. Frataxin is responsible for facilitating iron-sulfur clusters (ISCs) assembly in mitochondria, while PCBPs primarily bind to ferrous iron and transport it to ferritin and other non-heme iron enzymes, including deoxyhypusine hydroxylase, prolyl hydroxylase 2, and prolyl and asparagyl hydroxylases, through direct protein-protein interactions 23, 24. Additionally, PCBP1 can interact with BolA family member 2 (BolA2) and form a unique iron chaperone complex that transfers iron to the cell membrane for [2Fe-2S] cluster assembly, establishing a connection between the labile iron pool in the cell membrane and cellular ISCs distribution system 25. As a result, deficiency in both frataxin and PCBPs severely disrupts cell-autonomous iron homeostasis, leading to labile iron-induced ferroptosis 23, 24. Intriguingly, PCBP1 is also able to prevent ferroptosis triggered by ferritinophagy, a selective form of autophagy process that degrades ferritin, by functioning as an RNA-binding protein to destabilize beclin 1 (BECN1) 26. Notably, the iron chaperone and RNA-binding activities of PCBP1 are separate and occur at different sites, which independently confer essential functions 27. Therefore, a better understanding of the mechanism underlying the coordination of iron- and RNA-binding activities could provide valuable insights into the versatile roles of PCBP1 in diverse systems, including iron metabolism, immunity, cancer metastasis, and so on.

Excess free iron is stored in ferritin, a protein complex consisting of heavy-chain ferritin 1 (FTH1) and light-chain ferritin (FTL). The levels of ferritin subunits influence the rate of iron storage and the susceptibility to ferroptosis. Besides, overactive autophagy-lysosome-mediated degradation of ferritin and release of stored iron can lead to accumulation of labile iron, thereby triggering ferroptosis 28. During this process, nuclear receptor cofactor 4 (NCOA4) acts as a core adaptor to bind and deliver FTH1 to autophagosomes, which are then fused to lysosomes with the aid of vesicle-associated membrane protein 8 29. Thus, interfering with NCOA4-FTH1 interactions, such as FTH1 O-GlcNacylation 30, and the E3 ligase HERC2-mediated NCOA4 degradation, can impede ferritinophagy and subsequently ferroptosis 29. Recently, the vesicle trafficking gene SEC24B was identified as a novel driver of ferroptosis in microglia and macrophages 31. Despite of its known function in autophagosome formation, SEC24B did not affect ferritin degradation or ferritinophagy, but may regulate iron homeostasis by modulating the labile iron pool through potential mechanisms including altering export of iron transporters.

#### Iron export

Intracellular iron can be expelled from cells either by the membrane transporter FPN1, which is the most classical way, or by being transported out of the cell as ferritin via multivesicular bodies (MVBs). FPN1 is the only known mammalian iron transport protein, and thus it is conceivable that its expression and activity might affect iron metabolism and ferroptosis. In line with this idea, hepcidin, the only known natural ligand of FPN1 thus far, can bind to the central cavity between the N and C domains of iron-loaded FPN1 and induce ligand-receptor complex ubiquitination, internalization, and degradation to reduce iron output, increasing ferroptosis sensitivity 32. In contrast, ubiquitin-specific protease 35 maintains FPN1 protein stability by deubiquitination to prevent iron overload and ferroptosis 33.

Exosomes, a class of emerging extracellular vesicles (EVs) mediating inter-cellular and inter-organ communication, exist in various body fluids and are involved in numerous pathophysiological processes 34. Given that iron limitation inhibits ferroptosis, it is imaginable that the generation and secretion of ferritin-containing exosomes could attenuate ferroptosis. Indeed, prominin 2 (PROM2), a pentaspan membrane glycoprotein, can act as a ferroptosis braker by facilitating the formation of ferritin-containing MVBs and exosomal release of iron 35. Correspondingly, biocompatible hybrid nanoparticles composed of ferroptosis inducers and PROM2 siRNAs efficiently decreased iron efflux and overcome ferroptosis resistance in cancer cells, indicative of an attractive strategy for ferroptosis-based cancer therapy 36. Similarly, the inhibitors against heat shock factor 1, an activator of PROM2 transcription, can sensitize cancer cells to ferroptosis-inducing drugs and alleviate the oncogenic and ferroptosis resistance programs 37. Interestingly, CD63, a crucial regulator of exosome formation and secretion, is able to interact with NCOA4 and facilitate the exosomal export of iron, suggesting a role in ferroptosis 38. Moreover, CD63 has been shown to be post-transcriptionally regulated by iron via the iron-responsive elements/iron regulatory proteins system 38, adding an additional layer of complexity to the interplay between exosomes, iron efflux, and ferroptosis.

### Glucose metabolism

Glucose is the major carbon source for cellular biosynthesis and energy generation. Under aerobic conditions, acetyl-coenzyme A (acetyl-CoA), a key metabolite derived from glucose metabolism, enters the tricarboxylic acid (TCA) cycle and undergoes mitochondrial oxidative phosphorylation (OXPHOS) to generate highly efficient ATP. However, the process of OXPHOS is always accompanied by the production of toxic by-products such as superoxide anion radical, hydrogen peroxide, and hydroxyl radical, which are the main endogenous sources of ROS. In pancreatic ductal adenocarcinoma cells under high glucose conditions, SLC2A1-dependent glucose uptake and pyruvate oxidation-dependent fatty acid synthesis facilitate system Xc^-^ inhibitor-induced ferroptosis, while pyruvate dehydrogenase kinase 4 (PDK4), a suppressor of the conversion of pyruvate into acetyl-CoA that is downregulated by SLC2A1, can repress pyruvate oxidation, and thus resist ferroptosis 39.

Under conditions of limited oxygen supply, lactate dehydrogenase (LDH) facilitates the conversion of pyruvate to lactate, which appears to prevent the supportive effects of aerobic glucose oxidation on ferroptosis. Further compelling evidence reveals that hypoxia-inducible factor-1α (HIF-1α) can enhance LDH expression and lactate production, leading to pH-dependent resistance to ferroptosis in tumor cells 40. Moreover, heightened lactate uptake stimulates the generation of monounsaturated fatty acids (MUFAs) via activation of the sterol regulatory element-binding transcription factor 1 (SREBP1)/stearoyl CoA desaturase 1 (SCD1) axis 41. The replacement of PUFAs in cell membrane by MUFAs is able to mitigate lipid peroxidation and ferroptosis 42. Notably, lactate exists in two optical isomers, namely D-lactate and L-lactate. L-lactate is prevalent in mammals and serves as the primary carbohydrate fuel, signaling molecule for intercellular communication, and glycolytic precursor, but its implication in ferroptosis remains unknown. In contrast, the accumulation of D-lactate, whose concentration is very low in physiological serum, induced ferroptosis in tumor cells through increasing ROS and intracellular iron levels 43. Correspondingly, phosphorylation of Yes-associated protein (YAP) that enhances the transcription of D-LDH can facilitate D-lactate catabolism in mitochondria and safeguard tumor cells against ferroptosis.

The glucose pentose phosphate pathway (PPP) serves as a vital source of NADPH that assumes a dualistic role in ferroptosis, based on its capacity to supply electrons to various ferroptosis-regulating molecules and engage in interactions with functional proteins. NADPH actively engages in ROS detoxification through delivering electrons to antioxidant proteins including GSH, BH4, thioredoxin, and CoQ10, NAD(P)H quinone oxidoreductase 1 (NQO1) 44, 45. NADPH also supports the formation of ISCs and maintains iron homeostasis by providing electrons to ferredoxin2 46. However, it is worth noting that NQO1, in its interaction with the potential drugs like β-lapachone and 2-methoxy-6-acetyl-7-methylhomoquinone, can induce ROS production and drive ferroptosis, making it a target of interest in ferroptosis-focused cancer therapy 47. Furthermore, catalyzed by oxidoreductases such as NADPH-cytochrome P450 reductase (POR) and NADH-cytochrome b5 reductase (CYB5R1), electrons from NAD(P)H are transferred to oxygen, resulting in the generation of hydrogen peroxide that disrupts membrane integrity during ferroptosis 48. Additionally, NADPH is able to enhance the activity of the E3 ligase, membrane-associated ring-CH-type finger 6 (MARCHF6), which subsequently degrades of key ferroptosis inducers coenzyme A synthase long-chain family member 4 (ACSL4) and p53, and increases anti-ferroptosis proteins, including Nrf2, GPX4 and SLC7A11, thereby attenuating ferroptosis in cancer cells 49. Interestingly, MARCHF6 can mediate the ubiquitination and degradation of prohormone pro-opiomelanocortin (POMC), resulting in reductions in NADPH consumption by NOX2/NOX4, ROS production, and ferroptosis in POMC neurons 50. Altogether, these data suggest NADPH as a crucial intermediary mediator of ferroptosis, however its impact on ferroptosis is contingent upon specific substrates within a given microenvironment.

Additionally, a variety of metabolic enzymes related to glucose metabolism, such as ENO, creatine kinase B (CKB), myo-inositol oxygenase, and malic enzyme (ME), are involved in the regulation of ferroptosis. For example, all three of ENO isozymes, key glycolytic enzymes, are considered to be ferroptosis suppressors. α-enolase (ENO1), with a universal expression in adult tissues, can function as an RBP, beyond its metabolic activity, to degrade iron regulatory protein 1 (IRP1) mRNA, resulting in reduced expression of mitochondrial ferritin-1 (Mfrn1) and subsequent suppression of mitochondrial iron-triggered ferroptosis 4. γ-enolase (ENO2) and β-enolase (ENO3), which display tissue-specific expression patterns, have been implicated in ferroptosis 51, 52, however the underlying mechanisms are elusive and await further investigation. CKB, a key enzyme in ATP production, can phosphorylate GPX4 at serine 104 and block its degradation by heat shock cognate 70, thereby preventing ferroptosis 53. ME catalyzes the conversion of NAD(P)^+^ to NAD(P)H, and therefore deletion of ME1/2 reduces NADPH production, disrupting cellular redox homeostasis and increasing ferroptosis susceptibility 54, 55. Conversely, myo-inositol oxygenase, a non-heme ferritin that catalyzes the conversion of myo-inositol to D-glucuronide, is able to promote ferritinophagy-induced ferroptosis by reducing NADPH and GSH levels 56.

In summary, the metabolic enzymes, free radicals, and intermediate metabolites that are involved in the metabolism of glucose, the fundamental energy source of life, play a more expansive and versatile role in the process of ferroptosis, suggesting ferroptosis as an ancestral mechanism of cell death that accompanies both the beginning and end of life.

### Amino acid metabolism

Glutamine, glutamate, and arginine have been implicated in increasing sensitivity to ferroptosis. Inside cells, glutamine is deamidated by glutaminase (GLS) to produce glutamate, which is then converted to α-ketoglutarate (α-KG) by glutamate dehydrogenase 1. Glutamate and α-KG are the key intermediate metabolites in glutaminolysis-induced ferroptosis. Exogenous addition of glutamate can induce ferroptosis by inhibiting the uptake of cystine by system Xc^-^ 57. Elevated levels of endogenous glutamate result in increased cellular Ca^2+^ concentration, which subsequently phosphorylates and inhibits glutamine-fructose-6-phosphate transaminase (GFPT1) through activation of the adenylyl cyclase/protein kinase A axis. Decreased GFPT1 leads to destabilization of YAP, which reduces expression of its downstream target gene, FTH1, thereby reducing ferritin phagocytosis-triggered FTH1 transcriptional substitutions and enhancing sensitivity to ferroptosis 58. α-KG can serve as an intermediate in TCA cycle or be converted to 2-hydroxyglutarate by malate dehydrogenase 1, and thus trigger the generation of mitochondrial ROS (mtROS) and ferroptosis 59. Additionally, α-KG can activate p53-mediated ferroptosis by promoting oxidative DNA damage 59. Hence, glutaminolysis is considered a crucial contributor to ferroptosis, and it is plausible that inhibiting glutamine transporters like SLC1A5 and SLC38A1 or key nodes of glutaminolysis such as GLS could alleviate ferroptosis 57, 60. Argininosuccinate synthase 1 (ASS1) plays a role in reducing glutamine mitochondrial oxidative metabolism-induced ferroptosis by promoting reductive carboxylation of cytoplasmic glutamine 61. Moreover, ASS1 also facilitates the synthesis of arginine succinate, which is further cleaved to produce arginine. Arginine can activate the mTOR complex 1 (mTORC1)/SREBP1/SCD5 axis, which enhances the de novo synthesis of MUFAs utilizing acetyl-CoA derived from the glutamine reductive pathway and alleviates ferroptosis susceptibility.

Notably, arginine may also contribute to ferroptosis. Arginine has been identified as an essential amino acid for the induction of ferroptosis in HT1080 cells exposed to erastin2 or cystine deprivation 62. Furthermore, arginine has a role in sustaining fumarate biosynthesis through the urea cycle 63. Fumarate, as a reactive α, β-unsaturated electrophilic metabolite, can covalently binds to GSH, leading to a decrease in intracellular GSH levels and ultimately ferroptosis. This may be due to the fact that energy metabolism exhibits diverse trajectories in accordance with the requirements of vital biological processes, consequently yielding distinct consequences on ferroptosis. This is also evident in glutamine metabolism, as it contributes to the production of molecules like GSH (Glu-Cys-Gly) and NADPH that could protect cells against ferroptosis 64.

### Lipid peroxidation

Peroxidation of phospholipids containing PUFAs (PL-PUFAs) in cell membranes not only triggers liquid-liquid phase separation of the plasma membrane and impairs its physical and chemical functions, but also produces by-products such as 4-hydroxynonenal (4-HNE) and 4-hydroxyhexenal, which form adducts with biomolecules such as lipids, proteins, and DNA, ultimately resulting in ferroptosis 65. Among them, phospholipids containing a single polyunsaturated fatty acyl tail at the sn1 position of the glycerol backbone (PL-PUFA1s) have been extensively studied, whereas phospholipids possessing diacyl-PUFA tails at both the sn1 and sn2 positions (PL-PUFA2s) have received less attention. Recent investigations have demonstrated that an increased intake of PUFAs in the diet leads to an elevation in PL-PUFA2s, which interact with the mitochondrial electron transport chain to trigger mtROS generation and subsequent lipid peroxidation, thereby inducing ferroptosis 66. Furthermore, polyunsaturated ether phospholipids (PUFA-ePLs), particularly plasmalogens, owing to the presence of PUFAs at their Sn-2 position, can supply substrates for lipid peroxidation and facilitate ferroptosis induction 67. Therefore, regulating the content of PL-PUFAs is crucial in the regulation of ferroptosis. Diacylglycerol acyltransferase (DGAT) can facilitate the incorporation of PUFAs into the sn-3 position of diacylglycerol, resulting in the formation of triacylglycerol (TAG), which is stored as lipid droplets (LDs) surrounded by monolayers of phospholipids within cells. This process effectively decreases the level of PL-PUFAs. Inducing cell cycle arrest can enhance the DGAT-mediated reshuffling of PUFAs from phospholipids to TAG, consequently reducing the cell susceptibility to ferroptosis 68. In addition, the substitution of other types of lipids for PL-PUFAs undergoing peroxidation could attenuate ferroptosis. For example, 7-DHC, an intermediate in the cholesterol synthesis pathway, has recently been demonstrated as a natural inhibitor of ferroptosis 5, 6. Due to the unsaturated B-ring structure, 7-DHC can sequester free radicals and reduce lipid peroxidation of phospholipids, thereby safeguarding cells from ferroptosis. Ergosterol, an analog of 7-DHC, also exhibits ferroptosis-inhibitory properties 5, 6, suggesting that the trapping of free radicals to impede ferroptosis may be a shared chemical characteristic among these unsaturated sterols.

The initiation of lipid peroxidation requires the catalysis of a series of enzymes, including ACSL4, lysophosphatidylcholine acyltransferase 3 (LPCAT3), and ALOXs. Here, we specifically delve into novel insights regarding their potential relevance to endocrine metabolism. The involvement of the protein kinase C (PKC) family in metabolic diseases has been well-documented. Recent studies have revealed that PKCs also participate in promoting ferroptosis by activating lipid peroxidation enzymes. PKCβII was shown to phosphorylate the Thr328 residue of ACSL4, initiating the biosynthesis of PUFA-containing lipids and promoting lipid peroxidation 69. In turn, lipid peroxidation induces the activation of PKCβII, and thus amplifies ferroptosis through the lipid peroxidation-PKCβII-ACSL4 positive feedback loop. PKCα contributes to ferroptosis by phosphorylating the ALOX5 Ser663 70. LPCAT3 plays a crucial role in various processes including intestinal lipid absorption, hepatocyte lipoprotein assembly and secretion, adipocyte differentiation, and ferroptosis, due to its capability of incorporating unsaturated acyl groups into phospholipids 71. Interestingly, LPCAT3, also known as membrane-bound O-acyltransferase-containing structural domain (MBOAT) 5, belongs to the MBOAT family. Recent genome-wide CRISPR screening identified another two MBOAT members, MBOAT1 and MBOAT2, as ferroptosis suppressors 72. These two members can be transcriptionally induced by estrogenic and androgenic signaling, respectively, and mediate the effects of sex hormones on ferroptosis. Mechanistically, MBOAT1 and MBOAT2 hinder ferroptosis by selectively translocating MUFAs to the sn-2 position of phospholipids. Considering the significance of LPCAT3 and MBOAT1/2 in ferroptosis regulation, along with the similar enzymatic activities exhibited by other MBOAT family members, it is reasonable to speculate that other MBOAT members might also have regulatory effects on ferroptosis.

## The brakes of ferroptosis

**Figure 2:**
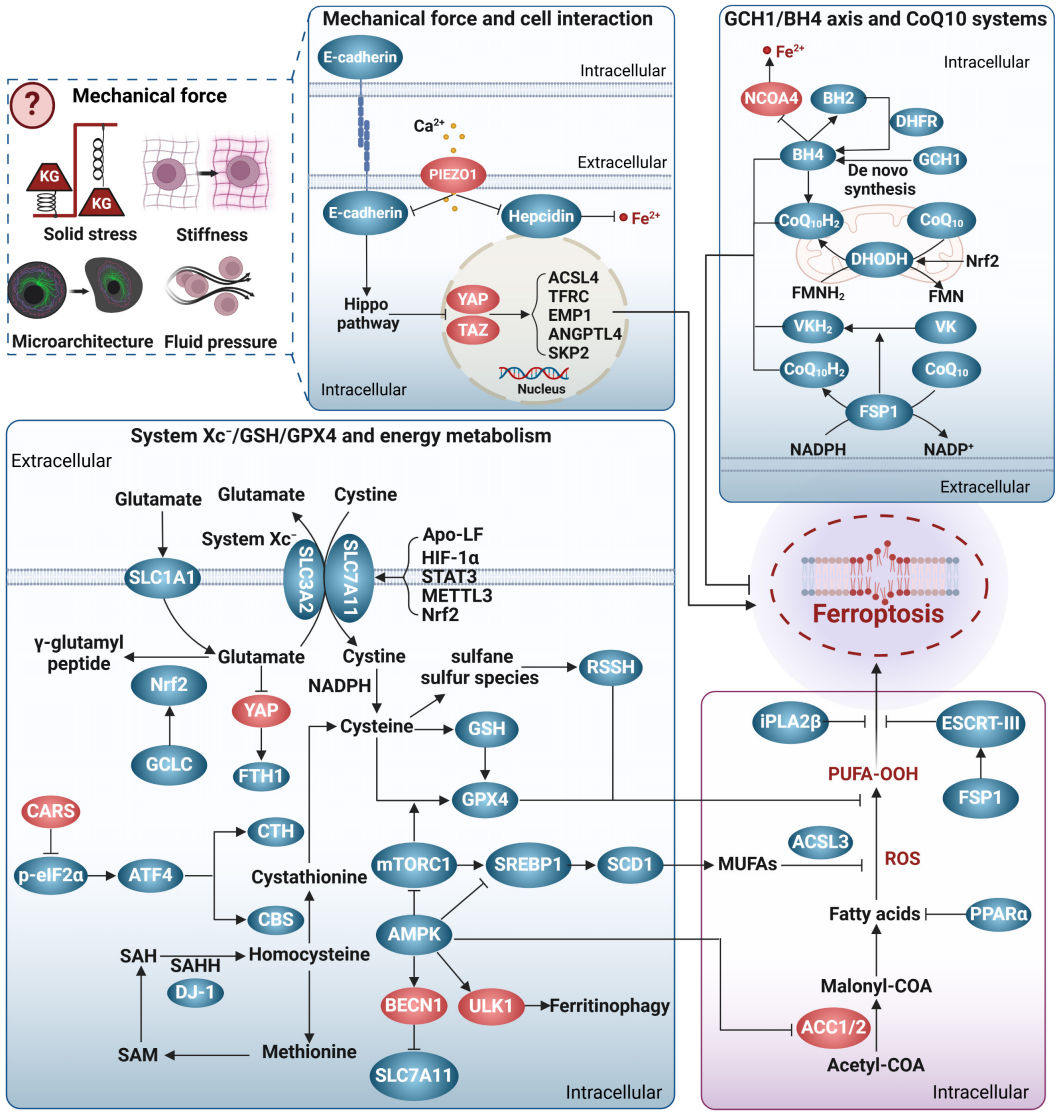
** Ferroptosis brakes.** The antioxidant system represents the most extensively investigated pathway that inhibits ferroptosis. This system encompasses the axis of system Xc^-^/GSH/GPX4, the GCH1/BH4 axis, and the CoQ10 system. The system Xc^-^/GSH/GPX4 axis is a well-known and classical anti-ferroptosis pathway, in which system Xc^-^ promotes the synthesis of GSH that in turn collaborates with GPX4 to convert lipid peroxidation products into alcohols, thereby effectively preventing ferroptosis. The transsulfuration pathway plays a role in providing cysteine for GSH synthesis, thereby conferring resistance against ferroptosis. The GCH1/BH4 axis, CoQ10 system, and VK also exhibit resistance against membrane phospholipid peroxidation through redox reactions. Energy metabolism regulatory systems, particularly AMPK and mTOR, also participate in the regulation of ferroptosis. Notably, AMPK displays a dual role in ferroptosis, which may be attributed to its diverse downstream targets. Interestingly, mechanotransduction appears to exert regulatory control over ferroptosis. Intercellular adhesion contacts can inhibit ferroptosis by activating the E-cadherin/Hippo/YAP signaling pathway. However, it remains unclear whether other forms of mechanical forces, such as solid stress, fluid pressure, stiffness, and physical microstructure, modulate ferroptosis. Arrows and vertical bars indicate positive and negative effects, respectively. Created with BioRender.com.

Cells have developed several efficient redox systems, including the system Xc^-^/GSH/GPX4, CoQ10, and GCH1/BH4 axis, which play crucial roles in cellular resistance to ferroptosis (**Figure 2**). Moreover, as imbalanced nutrient metabolism is related to induce ferroptosis, it is not surprising that cellular energy sensors such as AMPK and mTOR are involved in the regulation of ferroptosis. Additionally, it is important to recognize that cellular activities are intricately linked to mechanical signaling. Indeed, many mechanical checkpoints, such as integrin β1, focal adhesion kinase, YAP, and piezo-type mechanosensitive ion channel component 1 (Piezo1), have been shown to regulate ferroptosis.

### System Xc^-^/GSH/GPX4

System Xc^-^ is a cystine/glutamate transporter consisting of SLC7A11 and SLC3A2, which prevents ferroptosis by several ways. Firstly, it transports glutamate out of the cell, thereby preventing glutamate accumulation and subsequent ferroptosis 58. Secondly, it can supply cystine to the cell to support the synthesis of GSH, which cooperates with GPX4 in the reduction of the membrane phospholipid hydroperoxides to non-toxic lipids, thus protecting the cell from the ferroptosis caused by lipid peroxidation 73. Additionally, cysteine, the product of cystine reduction, serves as a source of sulfane sulfur in persulfides and polysulfides 74. Hydropersulfides (RSSH), in particular, exhibit even greater antioxidant capacity than alpha-tocopherol (the active ingredient in vitamin E), providing enhanced protection against ferroptosis 75. Thirdly, system Xc^-^ also participates in the transport of other amino acids, presenting an additional mechanism against ferroptosis. SLC7A11 is responsible for the transport of kynurenine into cell 76. Kynurenine is further metabolized to generate 3-hydroxykynurenine and 3-hydroxy-o-cyclo-anisoleic acid, both of which possess ROS scavenging and ferroptosis resisting activities 76. Interestingly, the competing use of SLC7A11 by kynurenine and cystine can be regarded as a temporary state of cystine deprivation or “pseudo-starvation”, necessitating the amplification of SLC7A11 transcription to fulfill the cellular requisites for amino acid absorption. This implies that the targeting of SLC7A11 has the capacity to concomitantly provoke nutrient deprivation and ferroptosis in energy-intensive tumors. Interestingly, p53, a frequently mutated tumor suppressor, is a repressor of SLC7A11 transcription. It has indicated that p53 mutation-triggered SLC7A11 overexpression can suppress ROS-induced ferroptosis and facilitate tumor metabolic reprogramming 77. Similarly, certain mutations in several genes, such as HJV, KDM5C, IDH1/2, and KRAS, have been linked to disease progression through modulating ferroptosis sensitivity and metabolism 78, 79. These observations suggest an emerging interplay among gene mutations, ferroptosis, and disease progression, which warrants further investigation.

The selenoprotein GPX4 is a pivotal enzyme in defense against ferroptosis. Nevertheless, metabolic disorders like selenium deficiency, copper accumulation, hyperglycemia, and hyperlipidemia have the potential to impact the expression and/or activity of GPX4, consequently leading to the induction of ferroptosis 80-83. More importantly, not all GPX4 isoforms possess the ability to inhibit ferroptosis. Depending on their cellular localization, three distinct isoforms of GPX4 exist: cytoplasmic (cGPX4), mitochondrial (mGPX4), and nuclear (nGPX4). Among them, cGPX4 is widely recognized as a key factor in protecting against iron-induced mutations 81. In cases where cells primarily experience damage from mitochondrial lipid peroxidation, the protective effect of mGPX4 surpasses that of cGPX4 84. nGPX4 is important for the integrity of sperm chromatin structure by modulating oxidation of cysteines in protamine 85. Interestingly, Xie *et al.* recently identified an inducible GPX4 transcript (iGPX4), whose expression was significantly increased in the livers of NAFLD. The induction of iGPX4 promotes the oligomerization of cGPX4, which counteracts the anti-ferroptotic properties of cGPX4 and induces ferroptosis, thereby aggravating NAFLD 81. Altogether, different GPX4 isoforms, together with their inter-translocation and interaction, greatly increase the complexity to dissect the role and mechanism underlying GPX4, and a better understanding would advance the GPX4-targeting strategies for ferroptosis and associated diseases.

### CoQ10 systems

CoQ10 plays a crucial role as a versatile electron carrier in supporting the mitochondrial respiratory chain. Additionally, reduced CoQ10 (CoQ10H2) functions as a potent lipophilic antioxidant, protecting against lipid peroxidation and ferroptosis 8. CoQ10 is synthesized within the inner mitochondrial membrane, which is typically impermeable. The dual localization of StAR-associated lipid transfer domain 7 (STARD7) in the cytoplasm and mitochondria enables it to regulate CoQ10 synthesis and intracellular distribution 86. Specifically, mitochondrial STARD7 ensures CoQ10 synthesis, while cytoplasmic STARD7 binds to CoQ10 and facilitates its passage through the mitochondrial membrane, thereby maintaining a balance between normal mitochondrial respiration and resistance against ferroptosis.

In the context of ferroptosis regulation, CoQ10 is primarily reduced to CoQ10H2 through ferroptosis suppressor protein 1 (FSP1)/CoQ10 and dihydronicotinic acid dehydrogenase (DHODH)/CoQ10 8, 84, 87. Recent studies have indicated that selenium can also generate selenides that enhance CoQ10H2 production via sulfide quinone oxidoreductase 88. The anti-ferroptosis effect of FSP1/CoQ10 or DHODH/CoQ10 has been demonstrated in various pathological models, and numerous proteins, non-coding RNAs, metabolites, and hormones have been identified as regulators of the FSP1/CoQ10 or DHODH/CoQ10 system 89-91. Specifically, both FSP1 and DHODH mediate NAD(P)H-dependent CoQ10H2 production in a similar manner, but their action sites differ, with FSP1 located at the cytosolic membrane and DHODH at the mitochondrial membrane 8, 84, 87. Subcellular localization is crucial for the anti-ferroptotic functions of FSP1 and DHODH. icFSP1, a new FSP1 inhibitor belonging to the 3-phenylquinazolinones class, has no role in FSP1 enzymatic activity, but induces FSP1 phase separation and leads to its separation from lipid membranes, thus hampering its anti-ferroptotic function 92. Interestingly, the interaction between FSP1 and 4-hydroxy-2-nonenal can relocate FSP1 from the mitochondria to the nucleus and promote apoptosis 93. Thus, proper subcellular localization appears crucial for FSP1 to exert specific biological functions. Whether the function of DHODH also exhibits site-selectivity remains unknown. Significantly, FSP1 also enables the reduction of vitamin K to hydroquinone, which effectively scavenges free radicals, underscoring the important role of FSP1 in supporting the antioxidant system against ferroptosis 94.

### GCH1/BH4 axis

BH4 is an effective endogenous antioxidant, and GCH1 is the main checkpoint for de novo synthesis of BH4. The GCH1/BH4 axis is suggested to be a predictor of ferroptosis sensitivity 9, and may protect cells from ferroptosis by at least two pathways. BH4 acts as a coenzyme to mediate the conversion of phenylalanine to tyrosine and increases the generation of 4-OH-benzoate, a precursor for CoQ10 biosynthesis 95. In this way, high levels of BH4 may promote de novo synthesis of CoQ10, and thus increase cellular tolerance to ferroptosis 9. Alternatively, the GCH1/BH4 axis may prevent ferroptosis via modulating iron metabolism. In a follow-up study, patients with phenylketonuria who responded to BH4 treatment had significantly lower serum iron levels, implying a role in iron homeostasis 96. Although the precise mechanisms underlying the regulation of GCH1/BH4 axis on iron metabolism are unclear, previous clues suggest that its inhibition could promote ferroptosis by activating ferritinophagy 97.

### Energy metabolism

AMPK and mTOR serve as crucial regulators of cellular energy status and play important roles in modulating ferroptosis. AMPK has been shown to prevent ferroptosis in conditions like diabetic cardiomyopathy (DCM), diabetic nephropathy (DN), NAFLD, and pulmonary fibrosis by promoting Nrf2 transcription or translation 83, 98, 99. AMPK also inhibits mitochondrial lipid peroxidation by activating forkhead box O 3a (FOX3A) signaling and blocking ACSL4 mitochondrial translocation, thereby reducing ferroptosis in cerebral ischemia-reperfusion injury and DCM 100, 101. Additionally, AMPK phosphorylates acetyl-CoA carboxylase 1/2 (ACC1/2) to inhibit fatty acid synthesis and reduce ferroptosis susceptibility 102. Nevertheless, recent evidence suggests a promotive effect of AMPK in ferroptosis. The synthesis of MUFAs is concurrently diminished by AMPK activation in certain conditions, which is not conductive to against ferroptosis 41. Consistently, AMPK induces ferritinophagy and subsequent ferroptosis by relieving inhibitory phosphorylation on unc-51 like autophagy activating kinase 1 (ULK1) 103. AMPK also phosphorylates BECN1 and facilitates the formation of the BECN1-SLC7A11 complex, thereby weakening the protective effect of system Xc^-^/GPX4/GSH 2.

mTOR exists in two multiprotein complexes, mTORC1 and mTORC2. In general, mTORC1 promotes anabolic processes and inhibits catabolism. To date, at least three pathways have been implicated in mTORC1-mediated ferroptosis repression. Firstly, mTORC1 can enhance the transcription, translation, and stability of regulators that resist ferroptosis, such as GPX4 104. Secondly, mTORC1 may protect against ferroptosis by promoting the synthesis of MUFAs via activating SCD 105. Thirdly, mTORC1 inhibits specific types of autophagy that contribute to ferroptosis 106. However, the degradation of cysteine-rich proteins such as albumin favors cellular cysteine replenishment, making the inhibitory effect of mTORC1 on autophagy unfavorable for ferroptosis resistance 107. Compared to mTORC1, the function of mTORC2 in ferroptosis is poorly understood. mTORC2 is predominantly activated by growth signaling and nutrient limitation 108. Recent studies suggest that the mechanisms by which cystine counteracts ferroptosis are partially attributed to mTOR2 activation 109. More specifically, Wang *et al.* demonstrated that mTORC2 can antagonize ferroptosis by enabling the nuclear translocation of Nrf2 through the activation of protein kinase B (AKT)/glycogen synthase kinase 3 β (GSK3β) signaling cascade 110.

In conclusion, while the roles of AMPK and mTOR in ferroptosis have been extensively explored, the complex disease background and the diverse repertoire of their downstream targets have complicated the establishment of a clear regulatory network linking energy metabolism and ferroptosis. Further research is required for a comprehensive understanding of the relationship between ferroptosis and energy metabolism.

### Mechanotransduction

Mechanosensory receptors within cells can detect mechanical stimuli and subsequently trigger signaling events that initiate biological responses. This process is known as mechanotransduction. Given the growing connection between metabolism and mechanical signaling, coupled with the recognition of mechanical signaling as a potential modulator of ferroptosis 111, a comprehensive investigation of ferroptosis and mechanotransduction might yield valuable insights into elucidating the interrelationship among these three factors.

In general, cells cultured at higher densities or in three-dimensional (3D) configurations exhibit enhanced resistance to ferroptosis, probably due to increased cell-cell interactions and activated cadherins 9, 112-114. Empirical evidence indicates that cadherin 2-mediated increased membrane tension can impede the process of ferroptosis 115. This inhibition potentially stems from the impact of heightened membrane tension on cell membrane fluidity, as well as the regulation of the uptake and release of iron and other extracellular metabolites. Furthermore, the mechanical activation of E/N-cadherin can initiate the Hippo signaling pathway, resulting in the inhibition of the YAP/tafazzin axis. This axis is known to promote the transcription of various genes associated with ferroptosis, including ACSL4, TFRC, epithelial cell membrane protein 1, angiopoietin-like 4, and E3 ligase S-phase kinase-related protein 2 112-114, 116, 117. Conversely, an elevation in hepatic extracellular matrix stiffness stimulates activation of the integrin β1/focal adhesion kinase/YAP pathway, consequently promoting ferroptosis 118.

Additionally, cell crowding, stretching and lipid peroxidation can modify membrane tension and activate mechanosensitive ion channels, such as Piezo1. Piezo1 acts as a homeostatic regulator for cell number and distribution by initiating cell death in crowded areas, but enhances cell division in sparse areas 119. Mechanistically, activation of Piezo1 leads to Ca^2+^ influx, which results in the degradation of VE-cadherin, reduced levels of GSH and GPX4, and increased cellular rupture, collectively intensifying ferroptosis 120-122. Interestingly, Piezo1 also exerts an inhibitory effect on heme expression in macrophages and hepatocytes, thereby exacerbating cellular iron accumulation 123, indicating that mechanotransduction has the potential to impact ferroptosis through the modulation of metabolism. Investigating the interconnectedness of mechanotransduction, metabolism, and ferroptosis may unveil a novel pathway for the regulation of ferroptosis.

## The cascade responses of ferroptotic cells

**Figure 3:**
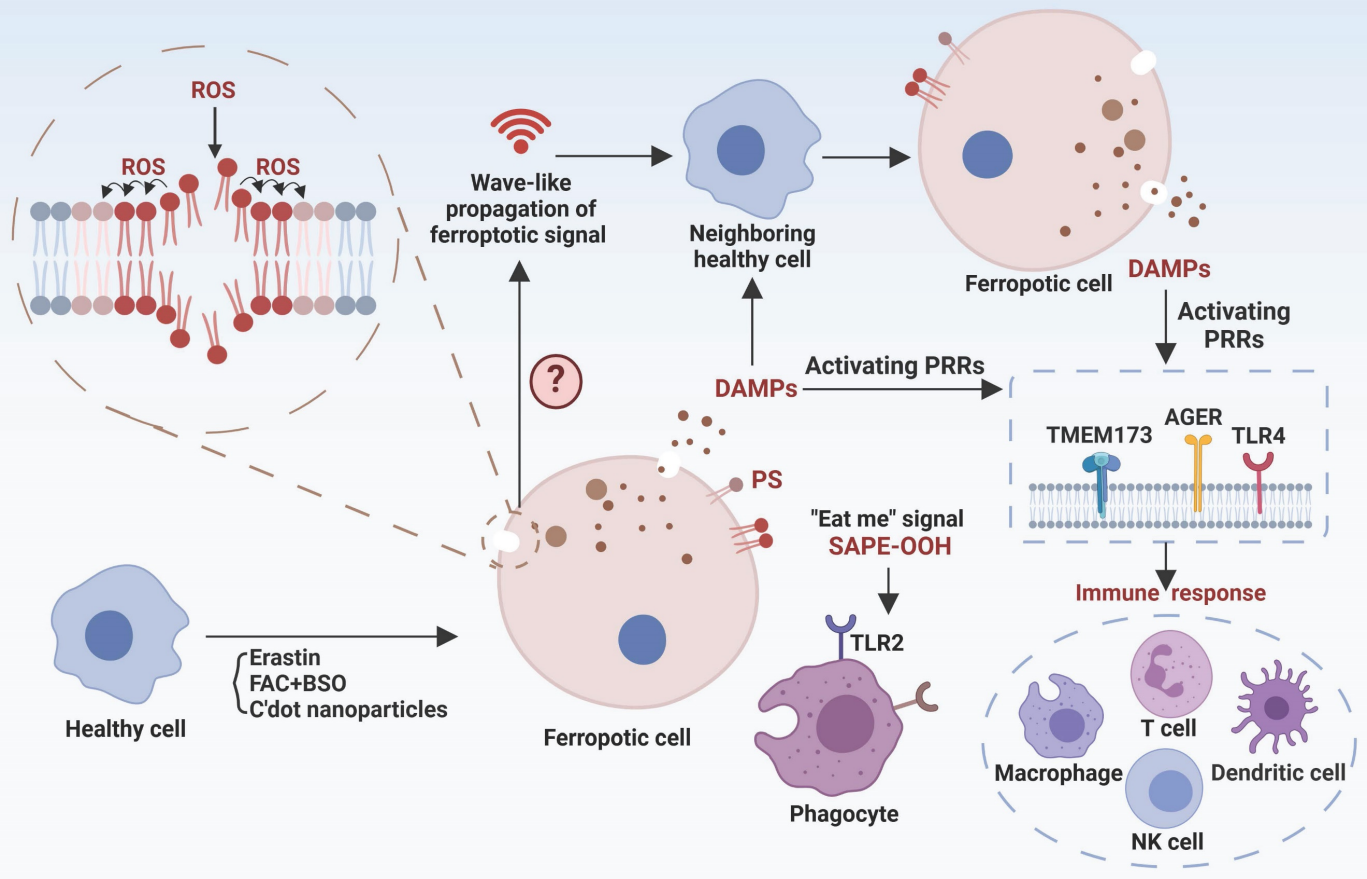
** The cascade reaction of ferroptotic cells.** Ferroptosis signals can propagate both intracellularly and intercellularly, thereby expanding the scope of ferroptotic processes. The lipid peroxidation signal associated with ferroptosis can propagate continuously across the cellular membrane. An undefined intercellular ferroptosis signal propagates in waveforms that may be linked to the ferroptotic cells released toxic substances. Furthermore, DAMPs are released through nanopores in membrane of ferroptotic cells and are recognized by PRRs, leading to the induction of immune responses, and ultimately sterile inflammation in metabolic diseases. Ferroptotic cells also have the capability to emit PS, SAPE-OOH, or other “eat me” signals that stimulate phagocytosis. Created with BioRender.com.

Ferroptotic signals in living organisms may continue with: (I) propagation of the death signal, (II) triggering immune responses, and (III) clearance by phagocytes (**Figure 3**), paving the way for the story of death.

### Ferroptotic death signal propagation

The dissemination of ferroptotic death signals has been observed in a wave-like manner throughout cell populations, influencing the fate of neighboring cells 124. The unique spatiotemporal ferroptotic signals occur prior to cell rupture, and osmoprotectant-mediated inhibition of cell rupture slows this propagation, suggesting a potential involvement of the "substances" in triggering ferroptosis. However, the exact definition of the ferroptosis signal remains unclear, including the identification of specific “toxic substances” and the selection of target cells to establish distinct spatial patterns. Hence, it is essential to identify ferroptosis signaling types and their mode of action for comprehending the progression of ferroptosis. Interestingly, platelet-activating factor (PAF) and PAF-like phospholipids, which are released by ferroptotic cells and actively internalized by adjacent cells via endocytosis, induce membrane rupture and subsequent cellular demise, exemplifying the propagation of ferroptotic death signals 12. EVs have recently been found to be an attractive transmitter for ferroptosis signals 125. For example, EVs derived from erastin-induced ferrototic cells can migrate to neighboring cells and subsequently expedite the death of these recipient cells. However, not all forms of ferroptosis exhibit this specific spatiotemporal propagation pattern. Particularly, cells undergoing ferroptosis induced by GPX4 inhibition exhibit a pattern of single-cell ferroptosis, wherein the death signal is not propagated to neighboring cells 124. In summary, the propagation of death signals is critical for biological, physiological, and pathogenic function of ferroptosis. Our current knowledge is almost certainly limited to the tip of the iceberg. Consequently, many exciting breakthroughs may lie ahead by using up-to-date technologies including single-cell omics, such as the discovery of potential systematic ferroptosis across distal tissues, the identification of spatiotemporal landscape and molecular mechanisms/checkpoints for ferroptosis propagation, and the dissection of different characteristics between single-cell and propagated multi-cell ferroptosis.

### Ferroptosis-related immune responses

Ferroptosis-related immune responses encompass both immune cell ferroptosis and immune disturbances provoked by ferroptotic cells. On one hand, immune cells exhibit varying susceptibility to ferroptosis, and ferroptosis in these cells directly influences the immune microenvironment and is associated with disease processes. For instance, aberrations in neutrophils are closely associated with various autoimmune diseases, and neutrophil ferroptosis significantly contributes to neutropenia in systemic lupus erythematosus, exacerbating disease manifestations 126. Antigen-specific memory CD4^+^ T cells play a crucial role in preventing microbial reinfection, and disturbances in their mTORC2 pathway result in aberrant accumulation of mtROS and subsequent ferroptosis 110. Therefore, triggering ferroptosis in antigen-specific memory CD4^+^ T cells have the potential to alleviate certain autoimmune conditions. Conversely, inhibiting ferroptosis to prolong the persistence of antigen-specific memory CD4^+^ T cells may contribute to the long-term efficacy of vaccination. Furthermore, dysfunction and depletion of CD8^+^ T cells play significant roles in tumor immune evasion. CD36 can facilitate fatty acid uptake, triggering CD8^+^ T cells ferroptosis and reducing their immunosuppressive effects on tumors 127. Interestingly, interferon-γ (IFN-γ) released by CD8^+^ T cells are able to downregulate the expression of two subunits of system Xc^-^, SLC7A11 and SLC3A2, while enhance ACSL4 expression in tumor cells, collectively promoting lipid peroxidation and ferroptosis 128, 129. Thus, combining induction of tumor ferroptosis with immunotherapy holds promise as a strategy for tumor management. Indeed, arsenic trioxide (ATO) is a potent broad-spectrum cytotoxic agent with highly immunogenic properties. ATO has the ability to convert immunologically “cold” tumors into “hot” tumors by enhancing the infiltration of immune cells and promoting the secretion of IFN-γ and tumor necrosis factor-α by CD8^+^ T cells. Simultaneously, ATO activates multiple cell death pathways, including ferroptosis 130. The development of an ATO-based cancer vaccine is anticipated to effectively prevent and intervene early in tumor progression. On the other hand, as ferroptotic cells release damage-associated molecular patterns (DAMPs), these molecules can bind to pattern recognition receptors (PRRs) on immune cells and alter their immune phenotypes. For example, DAMPs released by ferroptotic tumor cells can drive macrophage polarization towards the M2 phenotype in aggressive tumors, and ferroptotic neurons can activate CD4^+^ T cells, potentially promoting autoimmune encephalitis development 131, 132. Moreover, myeloid-derived suppressor cells (PMN-MDSCs), crucial negative regulators of antitumor immunity, are particularly vulnerable to ferroptosis in the tumor microenvironment. Although ferroptosis reduces the presence of PMN-MDSCs, the release of oxygenated lipids induced by ferroptosis limits the anticancer activity of T cells 133. Hence, ongoing efforts to translate pharmacological induction of ferroptosis in tumor cells into clinical practice should consider the impact of alterations in the immune milieu to ensure the specificity and minimal toxicity of the therapeutic approach.

Although recent studies have shed light on ferroptosis-associated immune disorders, it remains unclear whether immune cells have distinct mechanisms to sense, evade, transduce and release ferroptosis signals. Furthermore, current research focuses on ferroptosis-associated immune responses in tumors. Intriguingly, ferroptosis in immune or self-tissue cells has been shown to modulate the progression of autoimmune diseases 134, suggesting that autoimmune diseases may provide a valuable model for dissecting ferroptosis-associated immune responses.

### Ferroptotic cells clearance

Prompt identification and removal of ferroptotic cells are essential to reduce the propagation of ferroptotic signals and DAMPs. The “find me” and “eat me” signals exposed by dying cells interact with a series of receptors on the surface of phagocytes to mediate the phagocytosis of cell corpses. Phosphatidylserine (PS) and oxygenated PS exposed on the membrane are common “eat-me” signals involved in the phagocytosis of apoptotic cells. PS is also presented on the surface of ferroptotic Jurkat cells as a phagocytic signal, but its clearance efficiency is not as high as that on apoptotic cells 135. However, PS does not appear on the plasma membrane surface of ferroptotic HL60 cells and mouse embryonic fibroblasts. Therefore, different modalities of cell death may have their specific cell clearance signals. The oxidized phospholipid, 1-stearoyl-2-15-HpETE-sn-glycero-3-phosphatidylethanolamine (SAPE-OOH), has now been demonstrated to be a key “eat-me” signal of ferroptotic cells 136. Mechanistically, SAPE-OOH can directly interact with Toll-like receptor 2 (TLR2) of macrophages, resulting in effective elimination of ferroptotic cells 136. However, TLR2 deficiency was unable to abolish this elimination 136, suggesting alternative undocumented pathways. Notably, necroptic cells were found to evade phagocytic clearance by releasing “avoid me” signals, such as thromboxane that can disrupt macrophage OXPHOS and thus impair death cell clearance 137. It remains unknown whether ferroptotic cells have similar “avoid me” signals.

## Ferroptosis in metabolic diseases

**Figure 4:**
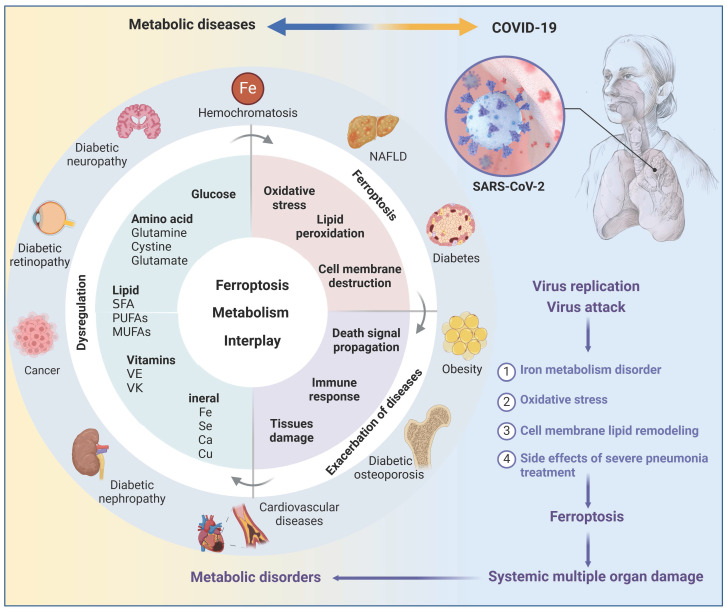
** The interplay of ferroptosis and metabolism in human diseases.** Disturbances in the metabolism of glucose, lipids, amino acids, and trace elements not only play a significant role in the pathogenesis of metabolic diseases such as obesity, diabetes, fatty liver, and hemochromatosis, but also serve as a crucial precondition for the occurrence of ferroptosis, suggesting a potential complementary relationship between metabolic diseases and ferroptosis. Metabolic dysregulation creates a favorable environment for ferroptosis, which further compromises tissue function and triggers inflammation, thereby exacerbating disease progression. It is crucial to note that obesity and diabetes are vital risk factors for COVID-19, and SARS-CoV-2 infection can induce ferroptosis. Thus, it is plausible that SARS-CoV-2 infection may cause metabolic imbalances in the microenvironment, subsequently trigger ferroptosis and result in secondary dysfunction across multiple organs. Created with BioRender.com.

Metabolic disorders always provide conducive conditions for ferroptosis, and ferroptotic cells may induce chronic inflammation and pathological cell loss that could exacerbate metabolic disorders, including obesity, diabetes and its complications, and NAFLD (**Figure 4**). Coronavirus disease 2019 (COVID-19), a disastrous pandemic, may also have a close correlation with ferroptosis. Untangling the intricate relationship between dysregulated metabolism, ferroptosis, and disease progression would facilitate the development of effective defense and therapeutic strategies.

### Obesity

Obesity, characterized by a body mass index greater than 30 kg/m^2^, is a prominent risk factor for various noncommunicable diseases, such as T2D, cardiovascular disease, and cancer. Recent research indicates that obesity can nurture an environment conducive to ferroptosis in the liver, kidney, and cardiovascular system, by promoting lipid peroxidation and iron overload 138, 139. Additionally, exosomes have an emerging role in obesity-associated ferroptosis, as exemplified by exosomal miR-140-5p derived from adipose tissue macrophages that induces ferroptosis in recipient cardiomyocytes by targeting SLC7A11 140. Paradoxically, obesity may confer a survival advantage to tumor cells by enabling resistance to ferroptosis. For example, adipose tissue-derived exosomes are enriched with microsomal triglyceride transfer protein (MTTP), which can increase GPX4 expression and decrease PUFAs levels, resulting in ferroptosis inhibition and drug resistance in colorectal cancer cells 141. In addition to exosomes, chemerin, a multifunctional adipokine, enables tumor cells to evade ferroptosis by inhibiting fatty acid oxidation 142. Of note, current research focuses on the impact of obese pathological environment on ferroptosis, but the role of ferroptosis in the development of obesity, such as adipocyte proliferation, differentiation, and function, remains to be fully understood.

### NAFLD

NAFLD has recently been redefined as metabolic dysfunction-associated fatty liver disease (MASLD), with a global prevalence of 30% and an increasing trend. Cell death, including necroptosis, pyroptosis, cuproptosis, and ferroptosis, have been reported in NAFLD, with ferroptosis being considered the primary cell death mechanism in early steatohepatitis 143. Two potent inhibitors of ferroptosis, namely Trolox (an antioxidant) and Deferiprone (an iron chelator), have demonstrated considerable potential in inhibiting cell death, inflammatory cytokine expression, and inflammatory cell infiltration in early stage non-alcoholic steatohepatitis (NASH) 143, highlighting the importance of ferroptosis in NAFLD pathogenesis and treatment.

Generally, NAFLD ferroptosis is closely associated with disrupted iron metabolism, and elevated serum ferritin levels is an independent predictor of advanced NAFLD 144, strongly suggesting a vicious cycle between ferroptosis and NAFLD. On the one hand, the microenvironment of NAFLD nourishes hepatic ferroptosis. As the main iron storage site, hepatocytes frequently experience disturbances in iron metabolism during liver injury. Meanwhile, hepatocytes often release excess iron to hepatic stellate cells via EVs, contributing to iron accumulation in these cells and the development of liver fibrosis 145. Moreover, metabolic dysregulation in NAFLD hepatocytes leads to the formation of iron pools that triggers Fenton reaction-derived ferroptosis 146. In addition, metabolites produced by the aberrant intestinal flora in individuals with NAFLD can also significantly induce the expression of TFRC and ACSL4 in the liver, enhancing the sensitivity of hepatocytes to ferroptosis 147. Notably, NAFLD progression is often associated with deregulated expression/function of key ferroptosis regulators, such as downregulation of Nrf2, GPX4, and SLC7A11, increased m^6^A methylation of SLC7A11, and hyperoxidization of peroxiredoxin 3 (PRDX3) 11, 98, 148, which collectively promote hepatocyte ferroptosis.

On the other hand, growing evidence emphasizes the significance of ferroptosis in NAFLD development. NAFLD encompasses a spectrum of conditions, ranging from simple steatosis and steatohepatitis to fibrosis. Diverse mechanisms linked to ferroptosis influence multiple pathological changes of NAFLD, including glucolipid toxicity, insulin resistance (IR), inflammatory cell infiltration, and hepatocyte loss. Therefore, inhibition of hepatocyte ferroptosis holds promise for attenuating the progression of NAFLD. Interestingly, ferroptosis in hepatic stellate cells seems to attenuate liver fibrosis 149. In this regard, a deeper understanding of the dynamics of ferroptosis and underlying mechanisms at different NAFLD stages and in distinct cell types is critical to advance the therapeutic use of ferroptosis in treating this disease.

### T2D

T2D accounts for nearly 90% of the estimated 537 million cases of diabetes worldwide and its prevalence is rapidly increasing. The pathological environment of T2D can promote ferroptosis through enhancing ferroptosis engines and diminishing ferroptosis brakes. For instance, hyperglycemia may increase glucose auto-oxidation and mitochondrial dysfunction in T2D, resulting in excessive ROS generation. Increased circulating fatty acids, a hallmark of obesity-associated T2D, heightens the risk of lipid peroxidation and subsequent ferroptosis 150. Moreover, T2D is associated with decreases in the expression and/or activity of various antioxidant enzymes, such as superoxide dismutase (SOD), GPX1, GPX4, thioredoxin reductase, thioredoxin reductase 2, glutathione, and peroxiredoxin 151. Dysregulation of AMPK expression and function may also contribute to T2D-associated ferroptosis 83.

Ferroptosis exerts a notable impact on numerous T2D pathological alterations, including β cell dysfunction, IR, inflammation and lipotoxicity. Ferroptosis is prevalent in β cells enriched with arachidonic acid (AA), which is crucial for insulin secretion, however, it increases susceptibility to lipid peroxidation 152. Experimental evidence underscores the significance of β cell ferroptosis in T2D, such as the impairment of glucose-stimulated insulin secretion 13. Conversely, certain compounds that attenuate β cell ferroptosis, including quercetin and cryptochlorogenic acid, exhibit potential in ameliorating T2D 153, 154. IR, a pivotal pathological hallmark of T2D, is partially due to ferroptosis. Inhibition of ferroptosis by liproxstatin-1 (a ferroptosis inhibitor) and ACSL4 knockout significantly alleviated hepatic and adipose IR, respectively 155, 156. Notably, ferroptotic cells typically release a range of signaling molecules and lipotoxic products, such as prostaglandin E2, 4-HNE, and hydroxyeicosatetraenoic acids, which might contribute to inflammatory cascades, impair the function of metabolic tissues, and stimulate angiogenesis, thereby triggering T2D complications 157. In conclusion, targeting ferroptosis may emerge as a promising strategy for impeding the progression of T2D through multifaceted mechanisms.

### Diabetic nephropathies (DN)

DN is the most serious microvascular complication of diabetes and the leading cause of chronic kidney disease. Glucose uptake by renal units is primarily regulated by ambient glucose levels, making the kidneys vulnerable to damage from high circulating glucose concentrations. At the same time, the kidneys are rich in mitochondria due to their high energy demands, which further increases their susceptibility to damage from ROS 158. Thus, there is an obvious elevation of ferroptosis in various types of renal cells under diabetic conditions.

A number of ferroptosis drivers and brakers that are deregulated in DN, together with various DN pathogenic factors, collectively modulate the initiation, progression, and elimination of ferroptosis. In cellular and animal models of DN, there is a decrease in ferroptosis suppressors (Nrf2, GPX4, SLC7A11, FTH1, etc.) but an increase in ferroptosis activators (ACSL4, TFRC, NOX1, PTGS2, etc.) 159. Besides, critical drivers of DN, such as transforming growth factor-β1 (TGF-β1) and HIF 1α, may induce ferroptosis in renal cells, and thus accelerate DN progression 160, 161. Specifically, TGF-β1 can repress GPX4 and SLC7A11 expression in renal tubular epithelial cells 160, whereas HIF1α is capable of activating heme oxygenase 1 (HO-1) that degrades heme and causes iron overload to promote lipid peroxidation and ferroptosis initiation 161. Notably, HO-1 may also function as a downstream target of Nrf2, thus exerting antioxidant effects in DN ferroptosis 162. In this regard, critical investigations using HO-1 deficient cell and animal models are required to clarify the functional outcome of HO-1 in DN development.

Consistently, ferroptosis presents a novel therapeutic avenue for DN. Numerous natural compounds, such as quercetin, ginkgolide B, and umbelliferone, have been extensively studied for their ability to enhance renal function and decelerate DN progression by attenuating ferroptosis 162-164. Further refinement of the chemical structures of these natural products to improve their targeting and specificity for ferroptosis may increase their translational efficacy of clinical interventions.

### Diabetic retinopathy (DR)

DR is a common microvascular complication of diabetes and is the leading cause of preventable blindness in the working-age population. Ferroptosis has recently been shown to be associated with the pathological process of DR. For instance, retinal pigment epithelial (RPE) cells play a crucial role in regulating the metabolic balance of retinal cell nutrients and maintaining the normal function of photoreceptor cells. Upon exposure to high glucose levels, extracellular glial maturation factor-β is increased and can induce ferroptosis in RPE cells by impeding autophagy-lysosomal degradation of ACSL4, contributing to the progression of DR 165. Correspondingly, 1,8-Cineole is able to alleviate DR by suppressing RPE ferroptosis 166. Moreover, high glucose levels may induce TRIM46 and promote GPX4 ubiquitination and subsequent ferroptosis in retinal capillary endothelial cells, exacerbating vascular permeability and capillary degeneration in DR 167.

### Diabetic cerebral vascular complications

Patients with obesity-related T2D exhibit excess iron deposition in the brain, which is associated with an elevated risk of stroke, cognitive impairment, and development of dementia 168. Hence, diabetes-induced neuronal ferroptosis may play a crucial role in the development of cerebrovascular complications associated with diabetes. Diabetic rats have reduced levels of FPN1 in the brain, leading to iron deposition and subsequent hippocampal ferroptosis that contributes to cognitive dysfunction 169. Diabetes also impairs the proliferator-activated receptor γ (PPARγ) signaling pathway in peripheral neutrophils, resulting in decreased LF transcription and secretion 170. This impairment contributes to iron deposition in neurons and, ultimately, neuronal ferroptosis. However, diabetic cerebrovascular lesions tend to have an insidious onset compared with other diabetic complications, emphasizing the significance of early detection and intervention. Advances in high-field magnetic resonance imaging techniques and reconstruction algorithms have shown promise in accurately mapping iron distribution, which contributes to the early diagnosis of diseases associated with ferroptosis and facilitates timely intervention 171. Significantly, caveolin-1 and hydrogen sulfide have been demonstrated to alleviate ferroptosis by reducing iron accumulation and oxidative stress in neurons and cerebral microvascular endothelial cells, and their effectiveness has been observed in reducing the susceptibility to diabetes-related cognitive dysfunction and psychiatric disorders, respectively 172, 173.

### DCM

Diabetes can cause alterations in the structure and function of the heart that raise the likelihood of developing heart failure. Glycolipotoxicity, mitochondrial dysfunction, and endoplasmic reticulum stress (ERS), have been identified as important contributors to the progression of DCM. Intriguingly, these mechanisms have also been implicated as significant pathways of cardiomyocyte ferroptosis in DCM, suggesting that controlling cardiomyocyte ferroptosis may be effective in delaying the onset and progression of DCM.

Specifically, in diabetic patients, increased insulin resistance causes a reduction in glucose uptake by the heart, leading to a shift towards utilizing fatty acids (FAs) as an energy source and advanced glycation end products (AGEs) accumulation 174. These factors are directly implicated in cardiomyocyte ferroptosis. Elevated uptake of FAs by cardiomyocytes not only induces lipid overaccumulation and the production of lipotoxic metabolites, but also exacerbates the accumulation of ROS within the cytoplasm and mitochondria, thereby fostering the development of ferroptosis in cardiomyocytes 150. Excess AGEs in cardiac tissues also elevate intracellular cytoplasmic ROS levels and subsequently trigger cardiomyocyte ferroptosis by downregulating SLC7A11 expression and reducing GSH levels 99. Additionally, endoplasmic reticulum (ER) has been identified as a significant site of lipid peroxidation in ferroptosis, and the quantity and functionality of ER may influence the vulnerability to ferroptosis 175. ERS-induced abnormalities in Ca^2+^ handling have been proposed as key factors contributing to DCM. Consistently, ERS appears to be a triggering factor for ferroptosis in cardiomyocytes during diabetic myocardial ischemia/reperfusion injury 176. Communications between the ER and mitochondria through mitochondria-associated ER membranes (MAMs) play crucial roles in Ca^2+^ signaling, energy metabolism, and cell survival. Dysfunction of MAMs has been implicated in the development of DCM 177. Interestingly, some molecular components of MAMs, such as voltage-dependent anion-selective channel 1 and mitochondrial protein 1/2, have been identified as important signaling molecules in ferroptosis 178, 179, suggesting that MAMs may represent an unexplored regulatory site for ferroptosis in DCM.

### COVID-19

There have been over 760 million recorded COVID-19 cases worldwide, and there is still limited knowledge about the pathological process and prognosis of SARS-CoV-2 on different tissues. The rising incidence of new-onset diseases, including diabetes 180, among individuals with COVID-19 underscores the significance of investigating the pathological alterations following acute COVID-19. Growing evidence suggests that SARS-CoV-2 has the ability to induce ferroptosis in host cells via various mechanisms 181, 182. Firstly, iron plays an important role in the replication of RNA viruses, including SARS-CoV-2. COVID-19 patients often have dysregulated iron metabolism, as manifested by elevated serum ferritin and hepcidin 183. Correspondingly, reducing iron levels not only curbs RNA virus replication, but also has beneficial immunomodulatory effects on the host and enhances antiviral capabilities 184. Secondly, hyperactive mitochondrial metabolic pathways are required to produce enough energy to support SARS-CoV-2 replication and reproduction, accompanied by excessive mtROS 185. Meanwhile, SARS-CoV-2 can inhibit the synthesis of some antioxidant enzymes, such as SOD3 and GPX4, in host cells, exacerbating the oxidative stress state 186. Thirdly, SARS-CoV-2 can reshape the cellular lipid membrane and promote the formation of replication organelle, providing the structural scaffold for the viral replication/transcription complex. Phospholipase A2 may participate in coronavirus replication organelle formation by cleaving the intact phospholipids on cellular and mitochondrial membranes to form lysophospholipids and unsaturated FAs 187. Besides, the levels of AA, palmitic acid and stearic acid are upregulated in COVID-19 individuals, which significantly increase the risk of lipid peroxidation and are associated with poor outcomes 188. These findings suggest that the disrupted metabolic environment triggered by SARS-CoV-2 may enhance vulnerability to ferroptosis in various organs. At the same time, ferroptosis could potentially serve as a trigger for the development of new-onset metabolic diseases in individuals affected by COVID-19 through various mechanisms, as mentioned in the T2D section. Therefore, it is reasonable to hypothesize that SARS-CoV-2 infection and ferroptosis form a vicious cycle to promote the progression of COVID-19 and its related metabolic diseases.

## Methods and prospects for ferroptosis detection

Reliable methods for accurate identification of ferroptosis amidst various modes of cell death are crucial for effective disease management. A combination of molecular biology, advanced imaging techniques, and biomedical engineering has been employed to develop a series of assays for detecting ferroptosis *in vitro* and *in vivo* (**Table 1**). As an easy means, transmission electron microscopy (TEM) is widely used to directly visualize the morphological changes of ferroptotic cells, such as mitochondrial condensation or swelling, increased membrane density, reduced cristae, and outer membrane rupture, thereby providing valuable insights into the ultrastructure of ferroptotic cells 175. However, these ultrastructural changes may occur in other types of cell death or cellular stress conditions. Therefore, the expression of ferroptotic signature genes, as well as the concentrations of certain metabolites, are always utilized to improve the detection of ferroptosis accuracy and dynamics. Due to the fact that ferroptosis is characterized by the peroxidation of PUFAs, liquid chromatography-mass spectrometry (LC-MS) is the preferred method for identifying peroxidized ferroptosis biomarkers. By combining MS-based lipidomics with proteomics, it is possible to characterize the ferroptotic covalent adducts of oxidative phosphatidylethanolamine (PE) with target proteins 189. However, LC-MS cannot offer information on the intracellular distribution of lipid molecules. Recently, gas cluster ion beam secondary ion mass spectrometry (GCIB-SIMS) that is capable of mapping intact lipids in cellular biofilms, coupled with a 70 keV (H_2_O)*_n_*^+^ cluster ion beam to amplify the signal, has enabled not only sensitive detection of intact biomolecules but also in-situ chemical imaging in ferroptosis cells 190.

Overall, MS-based assays provide high specificity and sensitivity for the detection and quantification of various molecules involved in ferroptosis, but they require specialized equipment and sample preparation. Moreover, when the natural cellular environment is disrupted, certain indicators of ferroptosis, such as Fe^2+^, can be rapidly oxidized and therefore cannot be reliably quantified from biopsy samples. As a result, a variety of small molecule fluorescent probes have been widely developed to enable real-time monitoring of ferroptosis-related indicators, including ROS, cysteine, GSH, lipid peroxidation, Fe^2+^, and Fe^3+^ (**Table 1**). For example, the redox balance of Fe^2+^ and Fe^3+^ is dynamic during ferroptosis, representing an attractive hallmark. Lu *et al.* recently developed DNAzyme-based fluorescent turn-on sensors that are selective for either Fe^2+^ or Fe^3+^ and can monitor both simultaneously, revealing a decreased Fe^3+^/Fe^2+^ ratio during ferroptosis 191. Additionally, specific fluorescent probes have been devised to assess the spatial, chemical, and physical microenvironments of ferroptotic cells, such as viscosity, polarity, and pH within subcellular organelles like the endoplasmic reticulum, mitochondria, and LDs (**Table 1**), enriching the landscape of evolving ferroptosis. Of note, due to the lack of a ferroptosis-specific biomarker, multifunctional fluorescent probes that can capture dynamic changes in various analytes are required to confirm and delineate the ferroptosis process. For instance, VPCPP, the near-infrared fluorescent probe that can simultaneously monitor local microviscosity, micropolarity, and carboxylesterase in living cells, revealed that sorafenib-induced ferroptosis led to an increase in the microviscosity and upregulation of carboxylesterase at the same time 192.

Nevertheless, most fluorescent probes for ferroptosis are limited by shallow tissue penetration depth, strong autofluorescence, and low signal-to-background ratios, which significantly impede their applications *in vivo*. Therefore, the development of sensors and imaging techniques that are more suitable for *in vivo* imaging is expected to facilitate the clinical translation of ferroptosis detection. Positron emission tomography (PET) and magnetic resonance imaging (MRI), two commonly non-invasive and quantitative imaging modalities for *in vivo* clinical testing, have been increasingly used for ferroptosis detection (**Table 1**). PET can provide quantitative 3D images for *in vivo* assessment of labile Fe^2+^ in ferroptotic cells 193, whereas MRI may offer advantages such as high resolution, absence of off-radiation, and deep tissue penetration, and is now more widely used in the diagnosis and treatment of ferroptosis-related diseases. For example, artemisinin-based probe (Art-Gd) is an MRI imaging probe specifically designed to target Fe^2+^ and exhibits promising capabilities for the early diagnosis of ferroptosis-related diseases. Building upon this, the POSS-Art-DOTA probe was developed, integrating PET and MRI to establish a dual-modality imaging technique that offers exceptional sensitivity and diagnostic precision 193, 194.

Despite of these advances, ideal approaches that can simultaneously detect multiple types of ferroptosis targets are still in their infancy. The combination of several advanced techniques, such as (H2O)_n_-GCIB-SIMS, C60-SIMS, desorption electrospray ionization, matrix-assisted laser desorption ionization, and artificial intelligence-assisted computational processing, has the potential to understand whether there are specific tissue compartments or cell types for ferroptosis, the metabolic landscape between these cells and the interconnected molecular networks between different cell populations. Improving the accuracy and safety of *in vivo* ferroptosis detection and accelerating its clinical translation would enable early detection of ferroptosis-related diseases and image-guided ferroptosis treatment.

### Ferroptosis-related therapy

**Figure 5:**
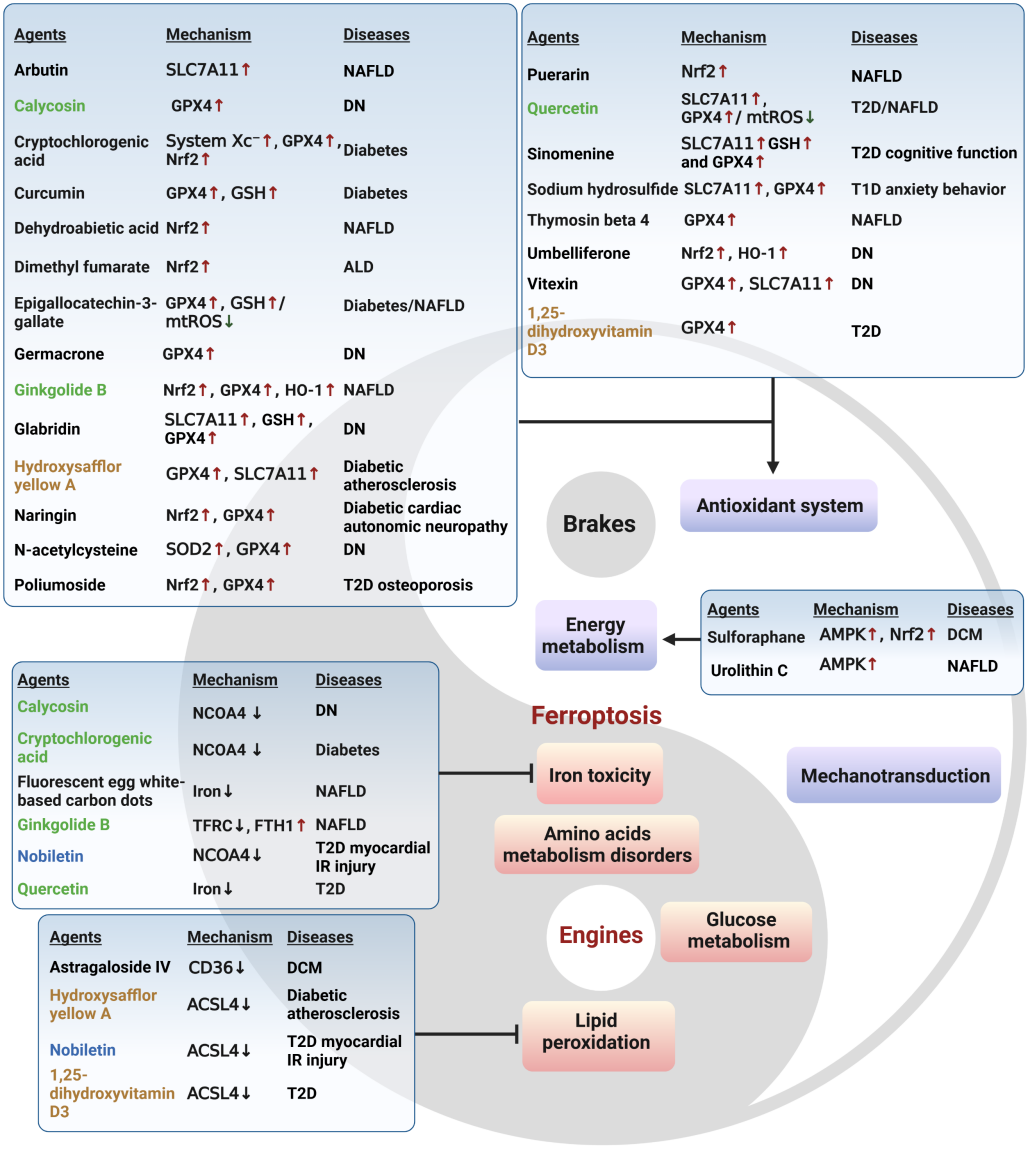
**Molecular compounds that ameliorate metabolic diseases by inhibiting ferroptosis.** Numerous natural products and experimental compounds have demonstrated efficacy in alleviating metabolic diseases by inhibiting ferroptosis, albeit its contribution and action mechanism are incompletely unknown. There is a summary of these molecular compounds targeting ferroptosis engines or brakes, potential mechanisms, and associated metabolic diseases. Compounds highlighted in green exhibit a dual function of acting as antioxidants and regulating iron levels. Those in blue have a dual function of inhibiting lipid peroxidation and regulating iron levels, while those in brown act as both antioxidants and inhibitors of lipid peroxidation. Arrows and vertical bars indicate positive and negative effects, respectively. Created with BioRender.com.

Targeting ferroptosis has emerged as a promising therapeutic approach. Multiple small molecule drugs have been shown to possess the capacity to modulate the previously mentioned “engines” or “brakes” of ferroptosis. These drugs can alleviate the ferroptosis-related physiological conditions like metabolic disorders (**Figure 5**), or combat tumor growth by inducing ferroptosis (**Table 2**). These studies with conventional drugs provide valuable information for the subsequent clinical translation of ferroptosis treatments. However, there are currently no clinical drugs specifically targeting ferroptosis, which is associated with the short history of ferroptosis research and the limited discovery of unique ferroptosis targets. Therefore, there is a pressing need to integrate new technologies to advance ferroptosis therapies, with nanomedicines showing significant promise.

There are various types of nanomedicines used to treat tumors by inducing ferroptosis, including drug delivery nanoparticles, active metal nanoparticles such as iron, manganese, copper and gold, single atom catalysts including selenium, nickel and iridium-based, multifunctional nanoparticles, and multifunctional magnetic nanomaterials 195-198. Among these, multifunctional magnetic nanomaterials have the ability to release magnetic iron oxide nanoparticles (IO) that can act as T1-weighted MRI agents upon reaching the tumor site, and thus induce tumor ferroptosis, enabling MRI-guided ferroptosis therapy.

For instance, IO-BQR@PMEMA exhibits a dark T2 signal in normal tissues, but when it reaches the tumor tissue, IO results in a bright T1 signal by the combined effect of ROS and GSH. Consequently, the released Fe^2+/3+^ and the drug Brequinar act synergistically to promote ferroptosis in tumor through Fenton reaction and DHODH inhibition, respectively 197. In conclusion, magnetic nanomaterials associated with ferroptosis offer new prospects for tumor therapeutics, and also provide a multi-scale for studying ferroptosis in the pathogenesis of diseases, such as metabolic disorders.

**Figure 6:**
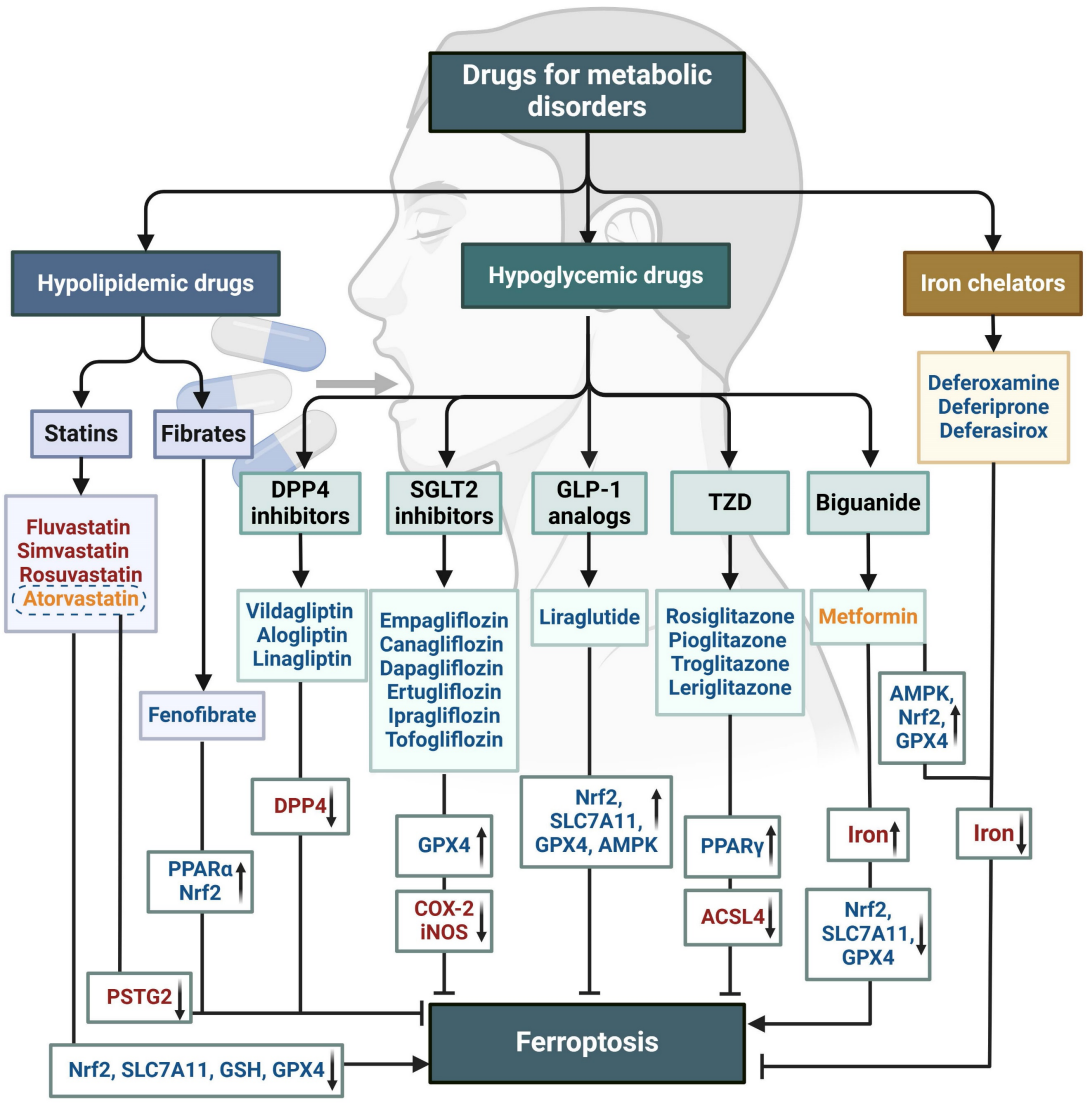
** The potential of approved drugs for metabolic disorders in modulating ferroptosis.** A range of drugs currently used in clinical practice for treating metabolic disorders, such as hypolipidemic and hypoglycemic drugs, as well as iron chelators, have demonstrated the ability to modulate ferroptosis. Drugs shown in blue inhibit ferroptosis, and those in red promote ferroptosis. Drugs in orange have a dual effect on ferroptosis depending on the disease context. Arrows and vertical bars indicate positive and negative effects, respectively. Created with BioRender.com.

The application of nanomedicines in treating metabolic diseases by restraining ferroptosis is relatively lagging behind. Only a few studies have reported on nanoparticles carrying natural products (such as curcumin and puerarin) or ferroptosis inhibitors (such as liproxsatin-1 and SOD) for the treatment of metabolic diseases like diabetic osteoporosis, fatty liver, and diabetic wounds 199-202. Significantly, while nanomedicines hold potential in the realms of disease diagnosis, treatment, and prevention, they are unlikely to swiftly supplant traditional medicines due to considerations surrounding *in vivo* absorption, metabolism, and long-term safety. Consequently, a prevailing approach during this transitional phase may involve the collaborative design and integration of traditional medicines with nanomedicines. A number of drugs approved for the treatment of metabolic diseases, including iron chelators, hypoglycemic agents, and hypolipidemic agents, have demonstrated significant efficacy in modulating ferroptosis in diverse disease conditions (**Figure 6**). Nanomedicines based on these drugs for treating ferroptosis in metabolic diseases have the potential to enhance safety, efficacy, and accelerate clinical translation.

## Conclusions

Metabolic dysregulation stands as a significant underpinning for ferroptosis development, which in turn exacerbates the progression of metabolic diseases through a cascade of reactions and cellular loss, perpetuating a detrimental cycle. Consequently, targeting ferroptosis has emerged as a novel strategy to impede the advancement of metabolic disorders. Nonetheless, numerous challenges persist in both mechanistic understanding and targeted therapeutics.

Numerous essential nutrients, such as glucose, FAs, glutamine, cystine, iron, and selenium, have been identified as crucial regulators of ferroptosis. However, the implications of various remaining nutrients, such as fructose, essential amino acids, and minerals (e.g. calcium, copper, and zinc), in ferroptosis remain unclear. Furthermore, the roles of key metabolic molecules (e.g. glutamine and NADPH) and regulatory nodes (e.g. AMPK) in ferroptosis regulation appear to be context-dependent, and their outcomes as either "engine" or "brake" of ferroptosis are frequently variable and await further dissection. Additionally, subcellular organelles including mitochondria, ER, and peroxisomes may serve as key sites for ferroptosis, but the mechanisms and outcomes of inter-organelle communication in orchestrating the progression of systematic ferroptosis are largely unknown.

The links between ferroptosis and the progression of metabolic diseases are widely acknowledged, the underlying mechanisms are incompletely known. To this end, further investigations into ferroptotic signaling pathways in diverse microenvironmental metabolisms are anticipated to provide clear and solid evidence. Intriguingly, the dysregulation of many hormones, such as insulin, glucagon, and sex hormones, is often observed in metabolic diseases, and emerging evidence indicates them as critical regulators of ferroptosis. For example, sex hormones and melatonin inhibit ferroptosis in tumor and neurons, respectively, while glucocorticoids can increase ferroptosis sensitivity. Therefore, it is of interest to investigate the potential of hormonal disorders in the synergistic development of metabolic diseases and ferroptosis.

Additionally, although the detrimental effects of metabolic dysregulation and ferroptosis on several systemic diseases are well documented, the involvement of these diseases in ferroptosis and metabolic disorders is emerging. For example, in aplastic anemia, ferroptosis of hematopoietic stem cells may exacerbate anemia and hypoxia, thereby disrupting systemic metabolic balance 203. Furthermore, prolonged transfusion therapy in such patients might cause hemochromatosis that could induce systemic iron overload and ferroptosis in hepatocytes and β cells, resulting in liver injury and diabetes. It is interesting for future research to expand the potential involvement of hematopoietic diseases in the pathogenesis of ferroptosis and metabolic diseases.

**Figure 7:**
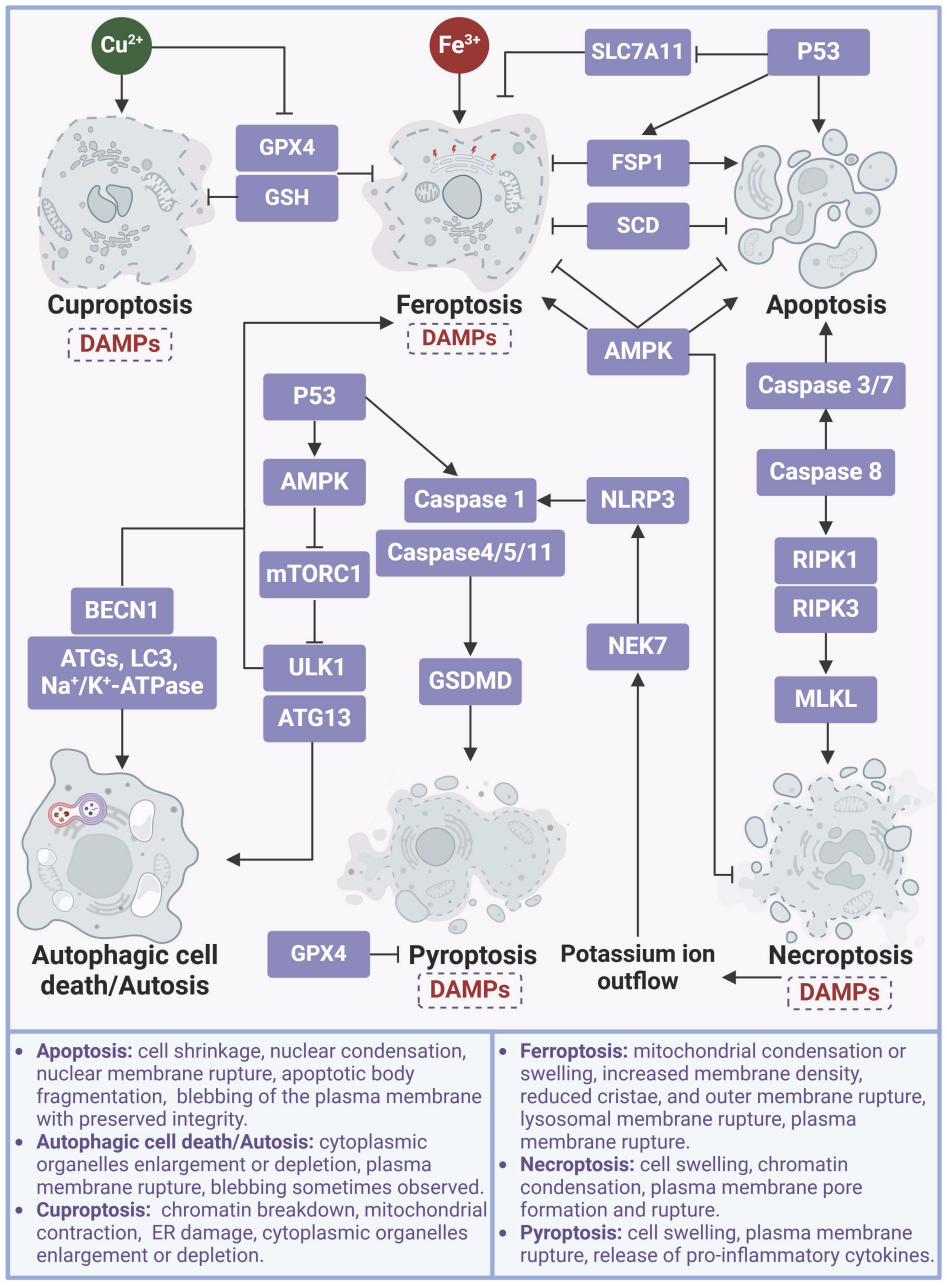
** Distinctions and interplay among cell death modes.** Different types of cell death, including apoptosis, necroptosis, pyroptosis, autophagy, cuproptosis, and ferroptosis, may simultaneously occur and interact in various physiological and pathological processes. Morphological changes of different cell death modes are also briefly summarized. Arrows and vertical bars indicate positive and negative effects, respectively. Created with BioRender.com.

Of note, different types of cell death, such as apoptosis, necroptosis, pyroptosis, autophagy, cuproptosis, and ferroptosis, may coexist and be coordinated in many physiological and pathological processes, adding another layer of complexity to ferroptosis research (**Figure 7**). Improvements on cell and animal models that mimic the sole and synthetical roles of distinct cell death modes, in combination with up-to-state technologies such as single-cell and spatiotemporal omics, could help to elucidate their entangled mechanisms and interplay, eventually enhancing our understanding of the trajectory of cell death during dynamic physiology and pathology.

## Figures and Tables

**Table 1:** Methods of ferroptosis detection

Targeting aspect	Specific materials		Targeting analyte	Technology/equipment
Morphology	/		Mitochondrial	TEM
	/		Lysosomal	
	/		Nuclear	
	/		Plasma membrane	
RNA expression of hallmark genes	Primers for corresponding genes		Hallmark genes	qRT-PCR
	/			RNA-seq
				scRNA-seq
				scRandom-seq
Protein expression of hallmark genes	Antibodies for corresponding proteins		Hallmark genes	Western blotting
				IHC
Oxidative lipidomics	/		PE	LC-MS/MS
	/		PEox	(H2O)_n_-GCIB-SIMS
	Probe	NBD-Pen	Lipid-derived radicals	LC/FL and HRMS/MS
ROS	Probe	Mito-QL and MBI-OMe	HOCl	Fluorescence microscope
		HP, BT-HP, QS-4 and BTFMB	H_2_O_2_	
		Coum-HCy	˙OH	
Biothiol	Probe	CP2 and Coum-HCy	Cysteine	
		CM-Mit	Mitochondrial thiol	
		RT	GSH	
Fe^2+^	Probe	FIP-1, Ac-MtFluNox, Lyso-RhoNox, ER-SiRhoNox, FeP and COU-LIP-1	Fe^2+^	
Heme	Probe	H-FluNox	Labile heme	
Fe^3+^	Probe	DRhFe	Fe^3+^	
Lipid peroxidation products	Probe	NBD-Pen, C11-BODIPY 581/591 and Liperfluo	Lipid peroxidation products	
pH	Probe	DSPI-3	pH	
Viscosity	Probe	DSPI-3, L-Vis-1, LD-1, Ir-ER, MN-V and BDHT	Viscosity	
Polarity	Probe	CQPP and TPA-SO2	Polarity	
Fe^2+^	Probe	POSS-Art-DOTA	Fe^2+^	PET/MRI
		Art-Gd		MRI

HOCl, hypochlorous acid; HRMS/MS, high-resolution tandem mass spectrometry; H_2_O_2_, hydrogen peroxide; IHC, Immunohistochemistry; LC/FL, online high-performance liquid chromatography-fluorometry; qRT-PCR, quantitative reverse transcription polymerase chain reaction; PEox, oxidized PE; ˙OH, hydroxyl radical; RNA-seq, RNA sequencing; ScRNA-seq, Single-cell RNA sequencing.

**Table 2:** FDA-approved drugs relevant to ferroptosis induction

Drugs	Target	Effects on ferroptosis	Clinical uses
Acetaminophen	Nrf2 inhibitor	Oxidative stress	Pain and fever
Cerivastatin	HMGR inhibitor	Inhibition of mevalonate synthesis	Hyperlipidemia
Dexamethasone	GSH inhibitor	Oxidative stress	Immunosuppressive therapy
Dimethyl fumarate	GPX4/GSH inhibitor	Oxidative stress	Multiple sclerosis
Fluvastatin	GPX4 inhibitor	Oxidative stress	Hyperlipidemia
Lapatinib	TF agonist; FPN1 inhibitor	Iron toxicity; Oxidative stress	Cancer
Leflunomide	DHODH inhibitor	Oxidative stress	Active rheumatoid arthritis
Lidocaine	SLC7A11 inhibitor	Oxidative stress	Heart disease
Metformin	SLC7A11 and Nrf2 inhibitor	Oxidative stress	Diabetes
Methotrexate	DHFR and GPX4 inhibitor	Oxidative stress	Cancer
Olaparib	SLC7A11 and AMPK/SCD1 inhibitor	Lipid peroxidation	Cancer
Orlistat	GPX4 inhibitor	Oxidative stress	Obesity
Regorafenib	System Xc^-^ and GPX4 inhibitor	Oxidative stress	Cancer
Simvastatin	GPX4 inhibitor	Oxidative stress	Hyperlipidemia
Sorafenib	System Xc^-^ inhibitor	Oxidative stress	Cancer
Sulfasalazine	System Xc^-^ inhibitor	Oxidative stress	CIRD; IBD

ARA, active rheumatoid arthritis; CIRD, chronic inflammatory rheumatic diseases; DHFR, dihydrofolate reductase; FDA, Food and drug administration; HMGR, 3-hydroxy-3-methylglutaryl-CoA-reductase; IBD, inflammatory bowel diseases.

## Abbreviations

AA: Arachidonic acid
Acetyl-CoA: Acetyl-coenzyme A
ACC1/2: Acetyl-CoA carboxylase 1/2
ACSL4: Coenzyme A synthase long-chain family member 4
AGEs: Advanced glycation end products
AKT: Protein kinase B
ALOXs: Arachidonic acid lipoxygenases
AMPK: Adenosine 5'-monophosphate-activated protein kinase
ASS1: Argininosuccinate synthase 1
ATO: Arsenic trioxide
BECN1: Beclin 1
BH4: Tetrahydrobiopterin
BolA2: BolA family member 2
cGPX4: Cytoplasmic glutathione peroxidase 4
CKB: Creatine kinase B
CoQ10: Coenzyme Q10
CoQ10H2: Reduced CoQ10
COVID-19: Coronavirus disease 2019
CYB5R1: NADH-cytochrome b5 reductase
DAMPs: Damage-associated molecular models
DCM: Diabetic cardiomyopathy
DGAT: Diacylglycerol acyltransferase
DHODH: Dihydroorotate dehydrogenase
DMT1: Divalent metal transporter 1
DN: Diabetic nephropathy
DR: Diabetic retinopathy
ENO: α-Enolase
ER: Endoplasmic reticulum
ERS: ER stress
EVs: Extracellular vesicles
FAs: Fatty acids
FOX3A: Forkhead box O 3a
FSP1: Ferroptosis suppressor protein 1
FTH1: Ferritin heavy chain
FTL: Ferritin light chain
FPN1: Ferroportin1
GCH1: GTP cyclohydrolase-1
GCIB-SIMS: Gas cluster ion beam secondary ion mass spectrometry
GFPT1: Glutamine-fructose-6-phosphate transaminase
GLS: Glutamate under the catalysis of glutaminase
GPX4: Glutathione peroxidase 4
GSH: Glutathione
GSK3β: Glycogen synthase kinase 3 β
HIF: Hypoxia-inducible factor
HO-1: Heme oxygenase 1
IFN-γ: Interferon-γ
iGPX4: Inducible GPX4 transcript
IONs: Iron oxide nanoparticles
IR: Insulin resistance
IRP1: Iron regulatory protein 1
ISCs: Iron-sulfur clusters
LC-MS: Liquid chromatography-mass spectrometry
LDH: Lactate dehydrogenase
LDs: Lipid droplets
LF: Lactoferrin
LPCAT3: Lysophosphatidylcholine acyltransferase 3
MAMs: Mitochondria-associated ER membranes
MARCHF6: Membrane associated ring-CH-type finger 6
MASLD: Metabolic dysfunction-associated fatty liver disease
MBOAT: Membrane bound O-acyltransferase domain containing
ME: Malic enzyme
Mfrn1: Mitochondrial ferritin-1
mGPX4: Mitochondrial GPX4
MRI: Magnetic resonance imaging
mTOR: Mammalian target of rapamycin
mTORC1: mTOR complex 1
mtROS: mitochondrial ROS
MUFAs: Monounsaturated fatty acids
MVBs: Multivesicular bodies
NADPH: Nicotinamide adenine dinucleotide phosphate hydride
NAFLD: Nonalcoholic fatty liver disease
NASH: Non-alcoholic steatohepatitis
NCOA4: Nuclear receptor coactivator 4
nGPX4: Nuclear GPX4
NOXs: NADPH oxidases
NQO1: NAD(P)H quinone oxidoreductase 1
Nrf2: Nuclear factor erythroid 2-related factor 2
NTBI: Non-TF-bound iron
OXPHOS: Oxidative phosphorylation
PAF: Platelet-activating factor
PCBPs: Poly rC Binding Proteins
PDK4: Pyruvate dehydrogenase kinase 4
PE: Phosphatidylethanolamine
PET: Positron emission tomography
Piezo1: Piezo-type mechanosensitive ion channel component 1
PKC: Protein kinase C
PL: Phospholipids
PMN-MDSCs: Myeloid-derived suppressor cells
POMC: Prohormone pro-opiomelanocortin
POR: NADPH-cytochrome P450 reductase
PPAR: Proliferator-activated receptor
PPP: Pentose phosphate pathway
PRDX3: Hyperoxidization of peroxiredoxin 3
PROM2: Prominin 2
PRRs: Pattern recognition receptors
PS: Phosphatidylserine
PTGS2: Prostaglandin endoperoxide synthase 2
PUFAs: Polyunsaturated fatty acids
PUFA-ePLs: Polyunsaturated ether phospholipids
ROS: Reactive oxygen species
RPE: Retinal pigment epithelial
RSSH: Hydropersulfides
TAG: Triacylglycerol
TCA: Tricarboxylic acid
TF: Transferrin
TFRC: Transferrin receptor
TGF-β1: Transforming growth factor-β1
TLR: Toll-like receptor
T2D: Type 2 diabetes
SAPE-OOH: 1-stearoyl-2-15-HpETE-sn-glycero-3 phosphatidylethanolamine
SCD: Steroyl CoA desaturase
SLC7A11: Solute carrier family 7 member 11
SOD: Superoxide dismutase
SREBP1: Sterol regulatory element binding transcription factor 1
STARD7: StAR-related lipid transfer domain containing 7
ULK1: Unc-51 like autophagy activating kinase 1
YAP: Yes-associated protein
3D: Three-dimensional
4-HNE: 4-hydroxynonenal
7-DHC: 7-dehydrocholesterol
α-KG: α-ketoglutarate
